# Diversity, Metabolic Properties and Arsenic Mobilization Potential of Indigenous Bacteria in Arsenic Contaminated Groundwater of West Bengal, India

**DOI:** 10.1371/journal.pone.0118735

**Published:** 2015-03-23

**Authors:** Dhiraj Paul, Sufia K. Kazy, Ashok K. Gupta, Taraknath Pal, Pinaki Sar

**Affiliations:** 1 Department of Biotechnology, Indian Institute of Technology, Kharagpur, 721302, India; 2 Department of Biotechnology, National Institute of Technology, Durgapur, 713209 India; 3 Department of Civil Engineering, Indian Institute of Technology, Kharagpur, 721302, India; 4 Central Headquarters, Geological Survey of India, Kolkata, 700016, India; University of North Texas, UNITED STATES

## Abstract

Arsenic (As) mobilization in alluvial aquifers is caused by a complex interplay of hydro-geo-microbiological activities. Nevertheless, diversity and biogeochemical significance of indigenous bacteria in Bengal Delta Plain are not well documented. We have deciphered bacterial community compositions and metabolic properties in As contaminated groundwater of West Bengal to define their role in As mobilization. Groundwater samples showed characteristic high As, low organic carbon and reducing property. Culture-independent and -dependent analyses revealed presence of diverse, yet near consistent community composition mostly represented by genera *Pseudomonas*, *Flavobacterium*, *Brevundimonas*, *Polaromonas*, *Rhodococcus*, *Methyloversatilis* and *Methylotenera*. Along with As-resistance and -reductase activities, abilities to metabolize a wide range carbon substrates including long chain and polyaromatic hydrocarbons and HCO_3_, As^3+^ as electron donor and As^5+^/Fe^3+^ as terminal electron acceptor during anaerobic growth were frequently observed within the cultivable bacteria. Genes encoding cytosolic As^5+^ reductase (*ars*C) and As^3+^ efflux/transporter [*ars*B and *acr*3(2)] were found to be more abundant than the dissimilatory As^5+^ reductase gene *arr*A. The observed metabolic characteristics showed a good agreement with the same derived from phylogenetic lineages of constituent populations. Selected bacterial strains incubated anaerobically over 300 days using natural orange sand of Pleistocene aquifer showed release of soluble As mostly as As^3+^ along with several other elements (Al, Fe, Mn, K, *etc*.). Together with the production of oxalic acid within the biotic microcosms, change in sediment composition and mineralogy indicated dissolution of orange sand coupled with As/Fe reduction. Presence of *ars*C gene, As^5+^ reductase activity and oxalic acid production by the bacteria were found to be closely related to their ability to mobilize sediment bound As. Overall observations suggest that indigenous bacteria in oligotrophic groundwater possess adequate catabolic ability to mobilize As by a cascade of reactions, mostly linked to bacterial necessity for essential nutrients and detoxification.

## Introduction

Arsenic (As) contamination in groundwater of alluvial aquifers worldwide creates serious problem in drinking water resources [1–4]. Among the contaminated sites, Bengal delta plain (BDP) that covers nearly 2 × 10^5^ km^2^ area of West Bengal (India) and Bangladesh, is the worst As affected geological province with more than 50 million people being under threat [4–6]. BDP is formed by the sedimentation of the rivers Ganges, Brahmaputra and Meghna, representing largest fluvio-deltaic basin (Holocene-Pleistocene alluvial deposition). Arsenic concentration in the solid phase of BDP aquifers is nearly consistent and represented mostly by As^5+^ [3]. It is found to be co-precipitated in or -adsorbed on various Fe and Mn rich clastic and authigenic minerals or mineral phases (Fe-hydroxides, Fe-oxides coated sand, phyllosilicates, Mn−, Al− oxides and hydroxides and authigenic pyrites) [3,7]. Aqueous As in contrast, is dominated by As^3+^ and its concentration showed high spatial and depth variations [8,9].

Extensive work carried out to identify the distribution pattern of As and its underlying geochemical mechanisms established that aqueous As is derived from solid phases due to complex interplay of biogeochemical processes between sediment-microbe-water systems [2,10]. In case of BDP, release of As is believed to be often closely related to release of Mn, but not with Fe and it is driven by microbial process that facilitate reduction of sediment associated elements (*e*.*g*., As, Fe or Mn) utilizing metabolizable carbon substrates [6]. Among the various mechanisms proposed, release of As due to weathering and/or dissolution of host minerals (Fe/Mn-oxides hydroxides) by complex geomicrobiolgical activities remains the most significant [11]. Biogeochemical activities of indigenous bacteria surviving under As rich, nutrient limiting aquifer environment can control mobility of this metalloid by transformation of host minerals or weathering of rocks [12,13]. Inspite of several confirmatory lines of evidences, our understanding about the identity of the indigenous bacteria and their metabolic properties relevant for As mobilization in groundwater remains least understood.

Microbial activity can lead to As release either by directly reducing the labile Fe^3+^/As^5+^ of minerals during anaerobic metabolism or indirectly by dissolution of nonlabile fraction of host minerals as a part of weathering mechanism [14,15]. In case of the later, As release occurs due to acquisition of essential nutrients followed by its redox transformation for detoxification or energetic gain. Several culture-independent molecular survey with anoxic incubations of As contaminated aquifer’s sediment or water have been revealed shift in bacterial community structures. Increased release of aqueous As with addition of carbon/electron sources was found to be coupled with predominance of Fe reducing *Geobacteriaceae* and sulphate reducing genera that transform As^5+^ via dissimilatory reductive processes [14,16,17,18,19]. Noticeably, several other studies on delineation of community composition revealed striking absence of dissimilatory As or Fe reducing organisms, but showed abundance of aerobic/facultative anaerobic, denitrifying, As transforming genera *e*.*g*., *Pseudomonas*, *Acinetobacter*, *Acidovorax*, *Hydrogenophaga*, *Brevundimonas*, *etc*. [9,10,20,21].

Arsenic contaminated aquifers of BDP and other areas of similar hydrogeology are often characterized to be oligotrophic in nature with low concentrations of organic carbon, O_2_, NO_3_^−^ and SO_4_^2−^ [3,4]. Lower concentrations of total organic carbon (TOC) in aquifer sediment and dissolve organic carbon (DOC) in the groundwater as observed in various studies implied presence of chemically mature system within the aquifer altered by microbial degradation processes (<1%) [22–25]. It has been hypothesized that both quantity and quality of labile organic matter and electron donor required for driving the As mobilization reactions are important for microbial action [15]. The combination of local hydrological and geochemical conditions and microbial processes that allow utilization of available organic carbons ‘foster the development of specific redox zones’ within the aquifer allowing mobilization of As [6,24]. Apart from organic carbon, the role of inorganic electron donors (*e*.*g*., As, Fe, Mn, *etc*.) in fuelling microbial metabolism towards As mobilization have been indicated by some investigators highlighting the presence and possible importance of chemolithiotropic processes in As contaminated BDP [15,26,27]. In recent years, there are some reports available about diversity and metabolic properties of inhabitant bacteria in As rich sites [27–32]. Presence of taxonomically diverse bacteria capable of heterotrophic as well as chemolithotrophic metabolism and redox transformations of As was frequently documented. Considering the nutrient limited nature of the aquifers, As transformation abilities have been linked to nutritional support (electron donor or acceptor) to the inhabitant bacteria as well as imparting changes to local geochemical conditions [14,29]. Several studies have reported the diversity and distribution of genes involved in resistance to As and its redox transformation within As rich aquifers and other impacted environments [10,30,33]. Among the various modes of As-bacteria interactions, preponderance of cytosolic As^5+^ reductase—As^3+^ efflux systems encoded by *ars*-*acr*(3) genes over the dissmilatory As^5+^ reductase (*arr*A gene) and periplasmic As^3+^ oxidase (*aio*B) genes was noted, particularly in As rich samples from gold mine, Andean salt deposits and geothermal geyser fields [10, 30,33]. Compared to these studies, bacterial diversity in As rich groundwater of alluvial aquifer of West Bengal and their functional abilities relevant for sustaining life under such oligotrophic environment, and in As mobilization from sediment have not been studied adequately.

In the present study we have explored bacterial diversity and community structure within six groundwater samples from two highly As contaminated sites of West Bengal (Barasat and Chakdaha) by both culture-independent and -dependent approaches. Metabolic characteristics including the genes involved in As homeostasis were investigated using culturable bacteria isolated from these samples. In order to gain insight into the underlying mechanism(s) of As mobilization by indigenous bacteria, selected culturable strains were used in anaerobic microcosms incubating them with orange sand. The orange sand was used in microcosm experiment due to its occurrence in Pleistocene aquifer which is less reduced, still anoxic and considered to be a potent source of low As and ‘safe’ groundwater. Tubewell systems based on these orange sand aquifers have been supplying drinking water to numerous villages of West Bengal but recent report on rise in As concentration in such wells showed their potential vulnerability.

## Materials and Methods

### Site description and sampling

Six arsenic contaminated groundwater samples (designated as AS1, AS2, AS3, AS9, AS40 and AS41) were collected from two most affected sites situated at Barasat and Chakdaha of North 24 Parganas and Nadia districts, respectively of West Bengal, during August, 2008 and January, 2009 (locations shown in Table 1). Samples were collected from household (domestic) tubewells. For sample collection no specific permission was required; however, the owners of the wells were informed about this work that is being carried out through our collaborators. The field study did not involve endangered or protected species. All the samples were collected in sterile 500 ml glass bottles after pumping the tubewells continuously for 15^_^30 min until the temperature (°C), electrical conductivity (EC), dissolved oxygen (DO), oxidation reduction potential (ORP) and pH readings got stabilized. These parameters were measured on site with portable Orion multiparameter meter (Thermo electron corporation, Beverly, MA) which was calibrated before the use. For microbiological investigation, sample bottles were stored immediately at 4°C prior to their analysis. For chemical analysis, samples were collected following the standard procedure that include filtering them through 0.45 μm filter membrane and acidification with trace element grade HNO_3_ (pH 2.0). Un-acidified filtered samples were collected for the analysis of major anions. The depth of the each tubewell (ranges 27–52 m) was noted from the record preserved by the well owners (Table 1). Arsenic containing orange sand used in this study was obtained from the inner part of the sediment core collected from Chakdaha block, Nadia district in West Bengal (location 23°01^′^049.00^″^N / 88°35^′^07.05^″^E) using a reverse circulatory drilling method done by Geological Survey of India, Kolkata. Samples were transported to the laboratory immediately after the collection and stored at 4°C prior to the use in our experiments.

**Table 1:** Geo-microbial properties of the groundwater samples.

Parameters	Sample ID					
	*AS1*	*AS2*	*AS3*	*AS9*	*AS40*	*AS41*
Block	Barasat	Barasat	Barasat	Chakdaha	Chakdaha	Chakdaha
Longitude	N22°44.062′	N22°44.056′	N22°44.064′	N23°01.235′	N23°01.214′	N23°01.215′
Latitude	E088°28.164′	E088°28.162′	E088°28.158′	E088°35.188′	E088°35.203′	E088°35.191′
Depth (m)	48.76	36.57	42.67	27.43	52.76	42.67
Temp (°C)	26.6±0.02	26.6±1.41	27.6±2.13	26.4±1.22	26.7±0.25	26.8±2.43
pH	6.78±0.001	6.84±0.99	6.47±0.21	6.75±1.3	6.63±0.70	6.85±1.2
Conductivity (μS cm^−1^)	673±2.03	847±3.45	668±9.21	1082±7.40	567±12.3	480±5.1
ORP (mV)	−82.8±0.87	−62.4±1.51	−18±1.31	−83.5±0.70	−86.9±1.1	−85.9±2.1
DO (mg L^−1^)	0.69±0.05	0.62±0.07	0.9±0.02	0.8±0.001	0.1±0.001	0.1±0.01
TOC (mg L^−1^)	4.7^e^±0.81	4.3^f^±0.06	4.1^d^±0.05	4.2^g^±0.05	3.8^e^ ^f^±1.11	3.6^h^±0.4
Total N (mg L^−1^)	11^d^±1.002	9.4^e^±0.29	5.04^d^±0.11	16.2^e^±1.10	8.1^d^±1.2	5.4^g^±1.5
Total P (mg L^−1^l)	0.35^h^±0.01	0.2^h^±0.001	0.64^e^±0.06	1.1^h^ ^i^ ^j^±0.001	0.15^g^±0.04	0.35^j^±0.10
Total S (mg L^−1^l)	1.87^f^ ^g^ ^h^±0.03	15^d^±1.00	0.63^e^±0.08	5^g^±1.05	0.75^f^ ^g^±0.08	32^b^±3.1
Fe (mg L^−1^)	12^d^±0.88	4.55^f^±0.01	0.67^e^±0.03	9.601^f^±1.21	4.605^e^±1.1	2.366^h^ ^i^±0.7
Mn (mg L^−1^)	0.903^h^±0.02	0.135^h^±0.03	1.095^e^±0.07	0.033^k^±0.003	0.81^f^ ^g^±0.75	0.426^j^±0.07
Ca (mg L^−1^)	88^c^ ±1.21	108^b^±1.10	88^c^±0.49	69.49^c^±4.55	72.72^b^±7.320	27.47^c^±2.5
Na (mg L^−1^)	3^f^ ^g^±0.05	3^f^±0.11	4^d^±0.81	9^f^±1.98	6^d^ ^e^±1.1	19^e^±1.20
K (mg L^−1^)	3.8^e^ ^f^±0.01	2.7^f^ ^g^±0.067	3.7^d^±0.48	2^h^±0.31	3^e^ ^f^ ^g^±0.95	1^i^ ^j^±0.64
As (μg ^L−1^)	518^a^ ±3.02	15^d^±1.22	1364^a^±17.03	440^a^±3.44	32^c^±2.54	22^d^±1.83
SO_4_^2−^ (mg L^−1^)	1.39^g^ ^h^±0.71	10.89^e^±1.39	0.846^e^±0.26	1.3^h^ ^i^±0.07	0.52^f^ ^g^±0.004	0.55^j^±0.006
NO_3_^−^ (mg L^−1^)	0.56^h^±0.03	0.06^h^±0.001	0.351^e^±0.021	0.03^k^±0.004	0.02^g^±0.001	0.02^j^±0.002
PO_4_^3−^ (mg L^−1^)	0.04^h^±0.001	0.03^h^±0.001	bdl	0.883^i^ ^j^ ^k^±.07	bdl	bdl
Cl^−^ (mg L^−1^)	5.367^e^±0.72	25.213^c^±1.76	4.727^d^±0.003	21.488^d^±2.01	8.345^d^±1.85	7.782^f^±1.08
F^−^ (mg L^−1^)	0.590^h^±0.01	0.930^g^ ^h^±.31	0.515^e^±0.024	0.520^i^ ^j^ ^k^±0.007	0.918^f^ ^g^±0.002	0.628^j^±0.10
HCO_3_^−^ (mg L^−1^)	360^b^±2.31	380^a^±1.34	376^b^±0.10	184.07^b^±10.8	147.2^a^±8.1	206.52^a^±7.21
NH_4_^+^ (mg L^−1^)	0.54^h^±0.034	0.27^h^±0.02	0.44^e^±1.02	0.140^j^ ^k^±0.001	0.76^f^ ^g^±0.003	0.16^j^±0.003
Microbial counts (CFU mL^−1^)	3.3×10^2^±0.4	2×10^2^±0.7	2.4×10^2^±0.2	2.8×10^2^±0.5	3.6×10^2^±0.9	3.4×10^2^±0.5
No of clones	153	136	210	189	85	163
Richness	17	18	19	31	11	21
Shannon (*H*)	2.2	1.9	1.9	2.6	1.7	2.3
Evenness (*E*)	0.78	0.66	0.65	0.76	0.7	0.75
Simpson,s (1/*D*)	5.9	4.3	3.9	8	4.2	6.2
Coverage (%)	98	93	99	95	97	96

bdl: below detection level

All data represent mean of triplicate (±) SD. Means followed by different letters are significantly different at the 0.05 probability level, grouped into classes a, b, c, d, e, f, g, h, i, j.

### Analytical techniques

Major anions (SO_4_^2−^, NO_3_^−^, PO_4_^3−^, HCO_3_^−^, F^−^ and Cl^−^) and cations (Ca^2+^, Na^+^, K^+^ and NH_4_^+^) in the water samples were estimated with an ion chromatograph (Dionex, USA). Total As, Fe and Mn were determined by atomic absorbance spectrometer (AAnalyst^TM^ 200, ParkinElmer, USA). Trace element concentration including As was measured with an inductively coupled plasma mass spectrometer (ICP-MS) (Varian 810 ICP-MS System, California). Total organic carbon, nitrogen, phosphorous and sulphur from the samples were assessed by following standard procedures as described APHA-AWWA-WPCF [34].

Mineralogy of the sediment sample (orange sand) was characterized by powder X-ray diffraction (XRD) using Panalytical high resolution XRD-I, PW 3040/60 diffractrometer (Almelo, Netherlands) with Cu-Kα1 radiation. Concentration of major and trace elements within the orange sand were determined by X-ray fluorescence spectrometry (PANalytical AXIOS, Almelo, Netherlands).

### DNA extraction, amplification of 16S rRNA gene and clone library preparation

Community metagenomes were extracted from all the water samples using water master DNA extraction kit (Epicenter, USA) following manufacturer’s instruction. PCR amplifications of bacterial 16S rRNA genes were performed using eubacterial forward primer 27F and universal reverse primer 1492R maintaining the reaction and temperature cycling conditions as described by Reardon et al. [35]. Details of the PCR conditions and primers used are presented in S1 Table. Purity of the amplified products (~1.5 kb) was determined by electrophoresis of 5 μl samples in a 1% agarose tris-acetate-EDTA (TAE) gel. DNA was stained with ethidium bromide and viewed under short-wave UV light.

For 16S rRNA gene clone library construction, amplified 16S rRNA genes were gel purified with Qiagen kit (Hilden, Germany), ligated into pGEM-T Easy vector (Promega, Madison, WI) and cloned into *Escherichia coli* JM109. Clone libraries were constructed with 100^_^200 randomly chosen white colonies per samples. Cloned 16S rRNA gene fragment from each positive colony was re-amplified using vector specific primers SP6 and T7. Amplified products were digested by restriction endonucleases (*Hae*III and *Msp*I) in separate reactions and were analyzed by 2.5% agarose gel electrophoresis. Amplified ribosomal DNA restriction analysis (ARDRA) patterns were grouped and each group was referred as an Operational Taxonomic Unit (OTU) or ribotype. At least one representative clone from each OTU was selected for sequencing of the 16S rRNA gene insert.

### PCR-DGGE analysis

V3 regions of 16S rRNA gene were amplified from metagenomes using GC-clamp 341F and 518R primers [36]. A DCode Universal Mutation Detection System (Bio-Rad, USA) was used to separate the PCR amplified 16S rRNA gene fragments by DGGE. Details of the primers used and PCR conditions are presented in S1 Table. A 8% (w/v) acrylamide solution (40% acrylamide bisacrylamide, 37.5:1 stock solution) in 1× TAE buffer with a denaturing gradient from 35 to 65% [100% correspond to 7 M urea and 40% (w/v) formamide] was used. Following electrophoresis in 1× TAE at a constant temperature of 60°C for 12 h at 70 V, gel was stained with ethidium bromide and was visualized under UV illumination. Distinguished thirteen bands in DGGE profile were carefully excised and eluted by keeping in 20 μl DNase free PCR water at 4°C. Those eluted products were re-amplified by using 341F and 518R primers and subsequently were cloned into the pGEM-T vector for sequencing.

### Enumeration, isolation and identification of bacteria

Aerobic heterotrophic microbial populations were enumerated by dilution plate count technique. For dilution plating, groundwater samples were serially diluted in normal sterile saline (0.9%) and 100 μl of suspension from each dilution was plated on R2A agar plate in triplicate and incubated at 26°C for 7 days. Number of bacterial colony forming units (CFUs) was counted at selected time intervals throughout the incubation period and morphologically distinct colonies were selected and purified by repeated subculturing in R2A medium. Each purified bacterial strain was stored at −80°C with 15% glycerol.

Taxonomic identification of isolated strains was done by analyzing the 16S rRNA gene sequences. Genomic DNA from each bacterial strain was extracted using genomic DNA isolation kit (Qiagen Ltd., Hilden, Germany) as per manufacturer’s instructions. PCR amplification of nearly complete 16S rRNA gene (1.5 kb) was carried out following the procedure described previously [35]. In brief, PCR amplification of 16S rRNA gene from the isolated genomic DNA was done using primer pair 27F and 1492R. Each 100 μl PCR reaction contained: 100 ng template DNA, 10 μl 10x reaction buffer, 5 μl MgCl_2_ (25 mM), 2 μl dNTP mix (10 mM), 10 mole of each primer, and 2U of Taq polymerase (Promega, USA). The PCR amplification was performed with an initial denaturation step of 94°C for 5 min; followed by 30 cycles of denaturation at 94°C for 60 s, annealing at 58°C for 45 s, elongation at 68°C for 90 s and a final elongation step at 68°C for 7 min. Amplified fragments were separated by 1% agarose gel electrophoresis and PCR products were purified by gel extraction according to manufacturer’s protocol (Fermentas, USA) and grouped together in phylotypes based on the RFLP analysis where two different restriction enzymes *Msp*I and *Hae*III were used [35]. At least one isolate from each RFLP group was identified by 16S rRNA gene sequencing.

### Arsenic sensitivity and metabolic characterization of the bacterial strains

Arsenic sensitivity and metabolic characteristics of bacterial strains isolated from six samples were studied using minimal salt medium (MSM) [37] with/without specific amendment(s) as required for the particular test. Arsenic sensitivity of the isolated strains was determined by growing the cells on MSM agar plates supplemented with 0.1^_^20 mM As^3+^ (as NaAsO_2_) or 1^_^100 mM As^5+^ (as Na_2_HAsO_4._ 7H_2_O) (Merck, Germany) diluted from 1 M respective stock solution under aerobic and anaerobic conditions. Mid log phase cells were inoculated onto the agar and the plates were incubated at 26°C for 7 days under aerobic condition. Plates incubated under anaerobic condition were amended with cystein hydrochloride (1g/l) and placed inside the anaerobic workstation for 14 days. As^3+^ oxidase and As^5+^ reductase activities of the isolates were tested using AgNO_3_ method described by Drewniak et al. [38], where reaction between AgNO_3_ and As^3+^ or As^5+^ formed coloured precipitate. A brownish precipitate was observed in presence of Ag_3_AsO_4_ (silver arsenate) and yellow precipitate in presence of Ag_3_AsO_3_ (silver arsenite). Siderophore formation was studied using Chrome Azurol S (CAS) agar media [39]. CAS agar plates were inoculated with bacterial strains and incubated at 30°C for 7 days. Colonies showing orange hollow zone following incubation were recognized as siderophore positive. Swimming motility was assessed following the procedure described previously by Lee et al., (2007) [40]. Where swim plates were made by adding 0.3% agar to R2A broth and inoculated with overnight grown bacterial culture. The plates were incubated at 30°C and bacterial migration was observed after 24 h. To check the carbon source utilization ability, MSM agar plate was amended with 10 mM of either of the following compounds (casein, glycerol, glucose, acetate, pyruvate, lactate, citrate, starch, sucrose and ascorbic acid). Metabolism of selected hydrocarbons (nonadecane, docosane, dodecane, pentadecane, cyclohexane, phenanthrene, naphthalene, pyrene, flurone and anthracene) was tested following bacterial growth in MSM broth where the test hydrocarbon (each 50 μg/ml concentration) was added as sole carbon source. All the hydrocarbons were obtained from Sigma (analytical grade). Cultures (plates and tubes) were incubated aerobically at 30°C to allow bacterial utilization of the test compounds and growth. Control plate or broth was made with MSM without any carbon source and incubated with bacterial strains under the same condition [41]. Utilization of alternate electron acceptor was assayed by allowing growth under anaerobic conditions in modified MSM agar plates supplemented with either of the test electron acceptors [As^5+^ (5 mM), Se^6+^ (5 mM), NO_3_^−^ (5 mM), SO_4_^2−^ (5 mM), Fe^3+^ (20 mM), S_2_O_3_^2−^ (5 mM) and SO_3_^2−^ (5 mM)] [42]. L-cysteine-HCl was added as a reducing reagent to quench the dissolved oxygen within the medium. Control plates were kept without addition of any electron acceptor and incubated with test strains under the same anaerobic environment. All anaerobic experiments were conducted within an anaerobic workstation (Coy laboratory products Inc.) filled with 95% N_2_:5% H_2_:5% CO_2_ mix gases. Chemolithoautotrophic property of the isolates was tested following their growth on M1 and M2 media [43] supplemented with 100 mM NaHCO_3_, vitamin (Composition mg/l: folic acid 12.5; riboflavin 25; thiamine 25; pantothenic acid 25; vitamin B_12_ 250; niacin 25; ascorbic acid 40) and trace elements (Composition g/l: EDTA 0.05; MgSO_4_.7H_2_O 3.00; MnSO_4_.H_2_O 0.5; NaCl 1.0; FeSO_4_.H_2_O 0.1; anhydrous CaCl_2_ 0.1; Al_2_(SO_4_)_3_ 0.01; H_3_BO_3_ 0.01; Na_2_MoO_4_ 0.01; Na_2_SeO_3_ 0.001), pH 7 to 7.2. One hundred mg/l of As^3+^ was added as electron donor in the test experiment to the earlier composition.

### Amplification of arsenic related genes

Genomic DNA of selected bacterial strains were used as template for PCR amplification of target As homeostasis genes (*viz*., *ars*C, *ars*B, *acr*3(2), *arr*A and *aio*B). PCR reactions targeting the *ars*C, *ars*B, *acr*3(2), *arr*A and *aio*B genes were carried out using primers and conditions as previously described [44–47]. Details of the PCR conditions and primers used are presented in S1 Table. The primer sets chosen here have been successfully applied in a variety of environmental samples. Selected amplicons of respective genes were individually gel-purified using the Qiagen kit (Hilden, Germany) (following the specified procedure) and quantified using nanodrop spectrophotometer (Thermo, USA). PCR products were ligated into pGEM-T Easy vector (Promega, USA), and cloned into *Escherichia coli* JM109.

### DNA sequencing and phylogenetic analysis

A total of 169 partial sequences (first 500–600 bp) of 16S rRNA genes from clones or isolated bacteria were obtained using an automated 3100 DNA sequencer. For each of our sequences, most similar sequences was retrieved from those available in public database by using the BLAST (NCBI) program (http://blast.ncbi.nlm.nih.gov/Blast.cgi) followed by initial classification using a web-based classifier program in ribosomal database project (RDP released 10 and with 95% of similarity) (http://rdp.cme.msu.edu/classifier/classifier.jsp). All the 16S rRNA gene sequences (retrieved from database and obtained in this study) were aligned by using ClustalW (http://www.ebi.ac.uk/Tools/msa/clustalw2/). Resulting alignments were used to construct the distance matrix followed by phylogenetic tree construction by neighbour-joining method using MEGA 4 software package [48]. Nucleotide sequences of As-resistance and As transformation genes were translated using the ExPASy tools (http://www.expasy.org/tools/dna.html), and appropriate reading frame for each gene was selected. Protein homology of translated products was determined using BLASTP of NCBI database. Phylogenetic trees were constructed using MEGA 4 with neighbour-joining method [48]. Bootstrap percentages (1,000 bootstrap replications) were used to test the robustness of phylogenetic relationships within the trees.

### Microcosm experiment

Arsenic bearing orange sand obtained from aquifer sediment was incubated anaerobically in artificial groundwater with selected strains (*Acinetobacter* BAS123i, *Arthrobacter* CAS4101i, *Brevundimonas* CAS4005i, *Pseudomonas* BAS323i, *Pseudomonas* CAS907i, *Phyllobacterium* BAS224i, *Staphylococcus* BAS108i and *Rhodococcus* CAS922i). Orange sand was thoroughly homogenised and sterilized by three rounds of autoclaving at 120°C, 15 psi for 40 min to remove the viable cells present within it. A total of nine microcosms, eight bioaugmented individually by eight individual bacterial strains and one control (without bioaugmentation by any bacteria), were prepared in triplicate using glass serum bottles (Sigma-Aldrich, St. Louis, USA). In each microcosm, 10 g of sterilized sand was added in 20 ml sterile artificial groundwater [15] amended with glucose and Na-acetate (10 mM each) as carbon source. In all biotic microcosms the initial bacterial cell density (added as inoculum) was maintained as 10^6^–10^7^ CFU/ml. All microcosm bottles were purged with ultrapure N_2_ gas for 2 hrs, sealed with butyl rubber stopper and incubated in the dark at 26°C over the entire period. Water samples (3 ml) were removed periodically from the bottles, centrifuged at 14,000 ×*g* for 5 min, and passed through a 0.45 μm membrane filter. The filtered but unacidified supernatant was used to determine concentrations of As^5+^ and As^3+^ by HPLC-HG-AAF (ParkinElmer, USA). Concentration of Fe^2+^ was measured spectrophotometrically using the ferrozine method [49]. Prior to analysis, the sample was completely solubilised by adding 1 ml of 0.5 N HCl to 50 μl of the desired sample and digested at 25°C for 24 h. Then Fe^2+^ was determined by adding 200 μl of sample digest to 1.5 ml of the ferrozone solution (1 g of ferrozine to 1 l of 50 mM HEPES buffer) and measuring absorbance at 562 nm. Total Fe was quantified using atomic absorbance spectrophotometer (AAS; AAnalyst 200, PerkinElmer, USA). Change in liquid phase arsenic and other element concentrations was measured by ICP-MS (Varian 810 ICP-MS System, California), as well as by AAS (ParkinElmer, USA) and/or Flame photometer (52A Flame Photometer Perkin-Elmer, USA) as appropriate. Organic acid concentrations present in the aqueous phase of microcosms were estimated by ion chromatography (Dionex, USA) using the procedure as describe by Frey et al. [50]. Change in sediment mineralogy before and after incubation with bacteria was tracked using XRD (Panalytical high resolution XRD-I, Almelo, Netherlands) and XRF (PANalytical AXIOS, Almelo, Netherlands) analyses. Colony forming units were determined at various time points using R2A agar medium and following the procedure as describe by Miles and Misra [51].

### Diversity indices and statistical analysis

For 16S rRNA gene clone libraries calculation of diversity indices and analysis of rarefaction were performed based on the number and frequency of ribotypes identified in each library. Shannon diversity indices (*H* and *E*) and reciprocal of Simpson’s index of dominance (1/*D*) were calculated following the standard procedure [35]. Percentage coverage of individual ribotypes in respective clone libraries was calculated using the equation C = 1-(n/N) x100, where n is the number of unique ribotypes and N is the number of sequences analyzed in the library [52]. Rarefaction curve was generated to compare the relative diversity and coverage of each library using the Analytical Rarefaction software version 1.3 (http://www.uga.edu/strata/software/index.html). Experimental observations were recorded with three replications (n = 3) and data were expressed as mean ± SD. The means were compared using analysis of variance (ANOVA) at 5% significant level (*p*≤0.05). One way ANOVA followed by Duncan’s multiple range tests was performed to determine the significant variations. Associations between variables were calculated by Pearson’s correlation. Relationship among the samples with respect to their geochemical properties was ascertained using Principle Component Analysis (PCA). Interrelationship between constituent bacterial populations and geochemical factors, in each sample was determined by Canonical Correspondence Analysis (CCA) using the XLSTAT (Addinsoft, New York, NY) package. Relationship among the samples with respect to cumulative metabolic properties of isolated bacteria was ascertained by Unweighted Pair Group Method with Arithmetic Mean analysis (UPGMA). Cumulative metabolic profiles of isolated bacterial strains were determined by calculating percentage value of positive response with respect to a particular metabolic parameter obtained within the isolates from individual samples (*e*.*g*., if all the isolates obtained from AS40 sample showed positive result for utilization of glucose as C (carbon) source, the value for AS40, glucose was computed as 100%). Interrelationship among the strains (selected) and their metabolic/genetic characters *viz*., ability to utilize different C sources and inorganic electron acceptors during anaerobic growth, chemolithiotropic growth, siderophore production and presence As transformation (*ars*C, *arr*A and *aio*B) and transporter [*ars*B and *acr*3(2)] genes were further determined by multivariate analysis using two component PCA. Data used in this PCA analysis were presented in S2 Table. In order to identify the relationship within bacterial As-resistance, As^5+^ reductase and As^3+^ oxidase activities and presence of As related genes UPGMA was done. MVSP 3.1 software was used for PCA and UPGMA analyses.

### Sequence data accession number

The sequences obtained in this study have been deposited in the GenBank database under accession no. KF379586^_^KF379706, KF442753^_^KF442789 and KF793264^_^KF793274.

## Results

### Physiochemical characteristics of groundwater

Physicochemical properties of the six groundwater samples were presented in Table 1. The samples were characterized by nearly neutral pH (6.5 to 6.9), low dissolved oxygen (0.1 to 0.9 mg/l) and negative ORP (^_^18.0 to ^_^86.9 mV). Among the anions and cations tested, concentrations of As, HCO_3_^−^, and Ca were found significantly (*p*≤0.05) higher. Arsenic concentration varied widely within the samples with lowest value (15 μg/l) obtained in AS2 and highest (1364 μg/l) in AS3. All the groundwater samples showed As level beyond the permissible limit for drinking water, *i*.*e*., 10 μg/l as set by World Health Organization [53]. Nevertheless, based on the concentration regime of test samples we categorized three of them (AS2, AS40 and AS41) as relatively low As (<50 μg/l) and remaining three (AS1, AS3 and AS9) as high As (>50 μg/l) containing samples. Concentrations of Na, K and Ca were nearly consistent within the samples while Fe and Mn showed considerable variation (0.67^_^12 mg/l Fe; 0.073^_^0.49 mg/l Mn). Among the trace elements Al, Ba, W and Zn were detected at relatively higher concentration ranges, *e*.*g*., 9^_^63 μg/l, 42^_^286 μg/l, 10^_^76 μg/l and 31^_^318 μg/l, respectively (S3 Table). Dissolve organic content was within the range of 3.6 to 4.7 mg/l. Culturable cell counts as enumerated using R2A agar medium showed 2×10^2_^3.6×10^2^ cells/ml of the samples. No significant correlation was found between the concentration of As and Fe in all the samples (R^2^ = 0.029, *p*≤0.05). Presence of total organic carbon (TOC) was found positively correlated (R^2^ = 0.4, *p*≤0.05) with As, while no significant correlation was observed between CFU and As or TOC (S4 Table).

### 16S rRNA gene clone library and community analysis

**Fig 1:**
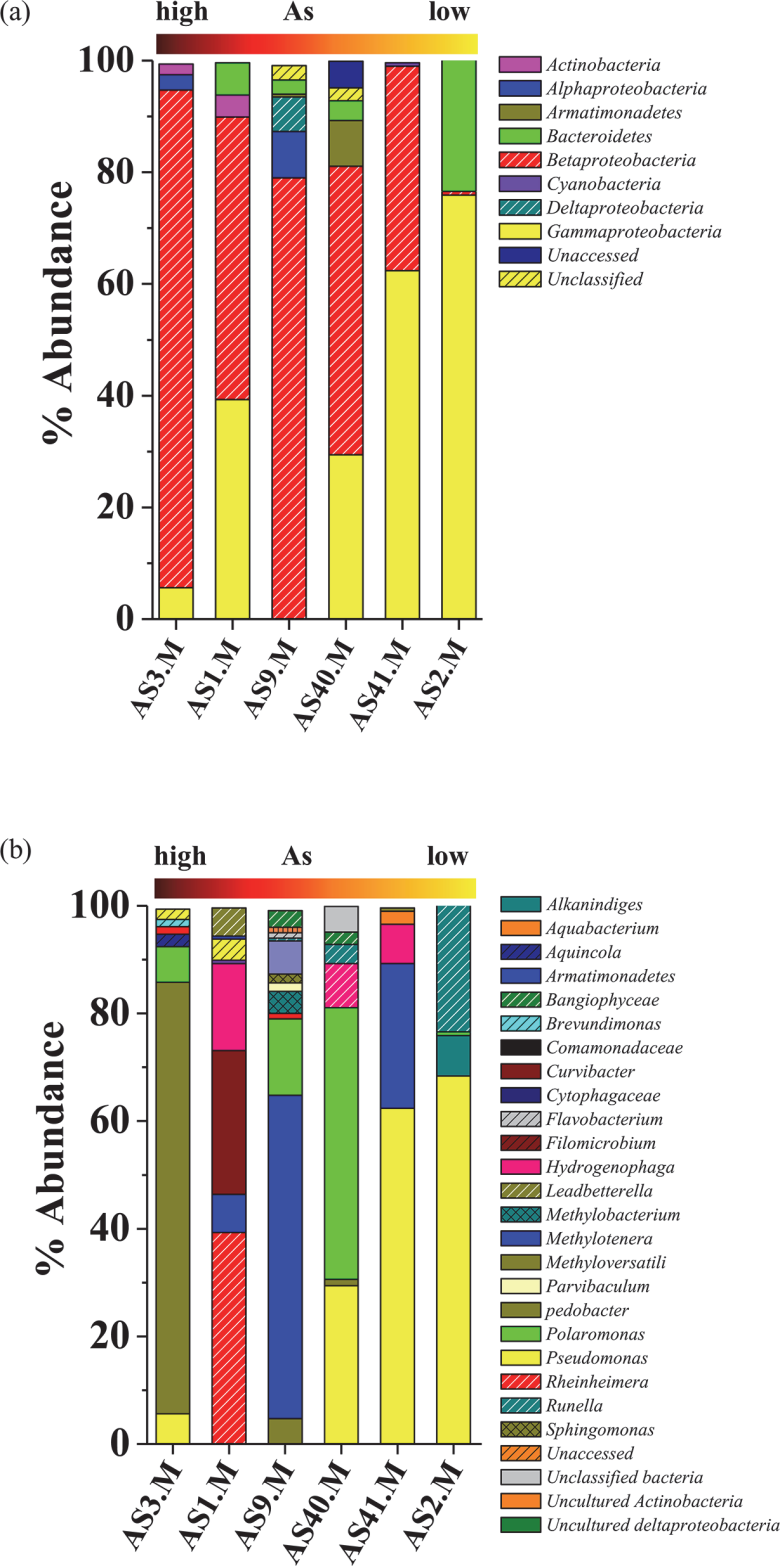
Distribution of major phylogenetic groups of bacteria as detected in clone libraries. Abundance of bacterial groups is plotted with respect to their affiliation at (a) phylum level and (b) genus level. The horizontal bar indicates gradient of As concentrations as detected in six samples.

Six clone libraries were constructed using the 16S rRNA gene amplified from groundwater community DNA and the clones were classified by ARDRA. Numbers of OTUs obtained from most of the libraries were within the range of 17^_^21 with number of clones ranging from 136^_^210. Goods’ coverage (>90%) and rarefaction data indicated high degree of clone coverage for the libraries (Table 1, S1 Fig.). Diversity index values (Table 1) showed considerably low diversity (H<3.0) with uneven community composition (E<1.0). Composition of bacterial communities was delineated by analysing 16S rRNA gene sequences of representative OTUs obtained from each library. It was noted that members of *β-* and *γ-Proteobacteria* were ubiquitously present within the samples followed by *Bacteroidetes* and *α-Proteobacteria* (Fig. 1A). Members of *δ-Proteobacteria*, *Armatimonadetes*, *Actinobacteria*, *Chlorobi* and *Cyanobacteria* were detected as relatively minor populations in a fewer samples. Presence of high GC containing bacterial groups *Bacteroidetes* and *Armatimonadetes* as well as *Cyanobacteria* and a few unclassified groups were observed in both high and low As samples.

Identification of bacterial groups at lower taxonomic level revealed presence of a number of genera (Fig. 1B). Genera *Pseudomonas*, *Rheinheimera*, *Polaromonas*, *Methyloversatilis*, *Methylotenera*, *Hydrogenophaga* of *γ-* and *β-Proteobacteria* and *Flavobacterium* (of *Bacteroidetes*) represented the major populations within the communities. Based on their ubiquity, bacterial genera detected here were grouped into two categories; one comprising of those detected in two or more samples (designated as frequently detected bacterial genera), while the other constituting genera found in only one of the samples (designated as less frequently detected bacterial genera).

### Frequently detected bacterial genera

**Fig 2:**
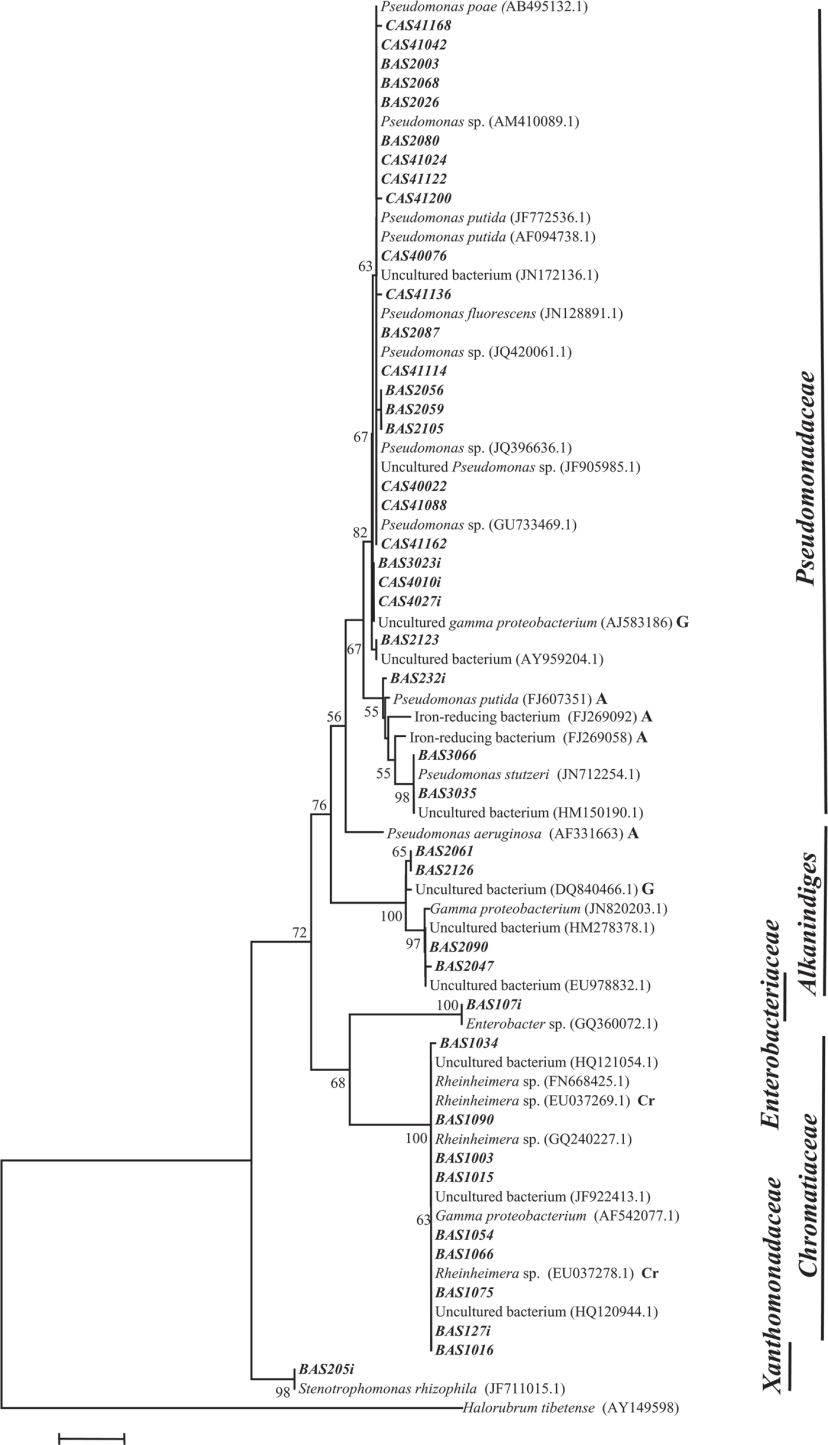
Neighbor-joining phylogenetic tree of *γ-Proteobacteria*. The tree is constructed based on 16S rRNA gene sequences by using Jukes-Cantor distances. 1000 bootstraps analyses are conducted and more than 50% are denoted in nodes. Sequences retrieved from As contaminated environment, groundwater and chromium contaminated sites are suffixed here as ‘A’, ‘G’, and ‘Cr’, respectively. Sequences represented in bold italic font are derived from clone libraries and bold italic font with a suffix ‘*i*’ are derived from isolated strains.

Genus *Pseudomonas* represented the most abundant population (29^_^68%) in all three low As samples as well as a minor population (5.6%) in the highest As containing sample (AS3). Phylogenetic analysis with often similar sequences showed that Pseudomonad related sequences segregated into two clades (Fig. 2). Sequences obtained from the low As samples were showed lineages to various cultured and uncultured *Pseudomonas* species reported earlier from rhizosphere and other subsurface environments. Absence of similarity was found between these sequences with other *Pseudomonas* sequences retrieved previously from As contaminated sites or aquifers. In contrast to this sequences retrieved from AS3 showed similarity and close lineage with cultured *P*. *putida*, *P*. *stutzeri* and uncultured Fe reducing *Pseudomonadace* bacteria previously obtained from As contaminated aquifers of Bangladesh and other As rich sites.

**Fig 3:**
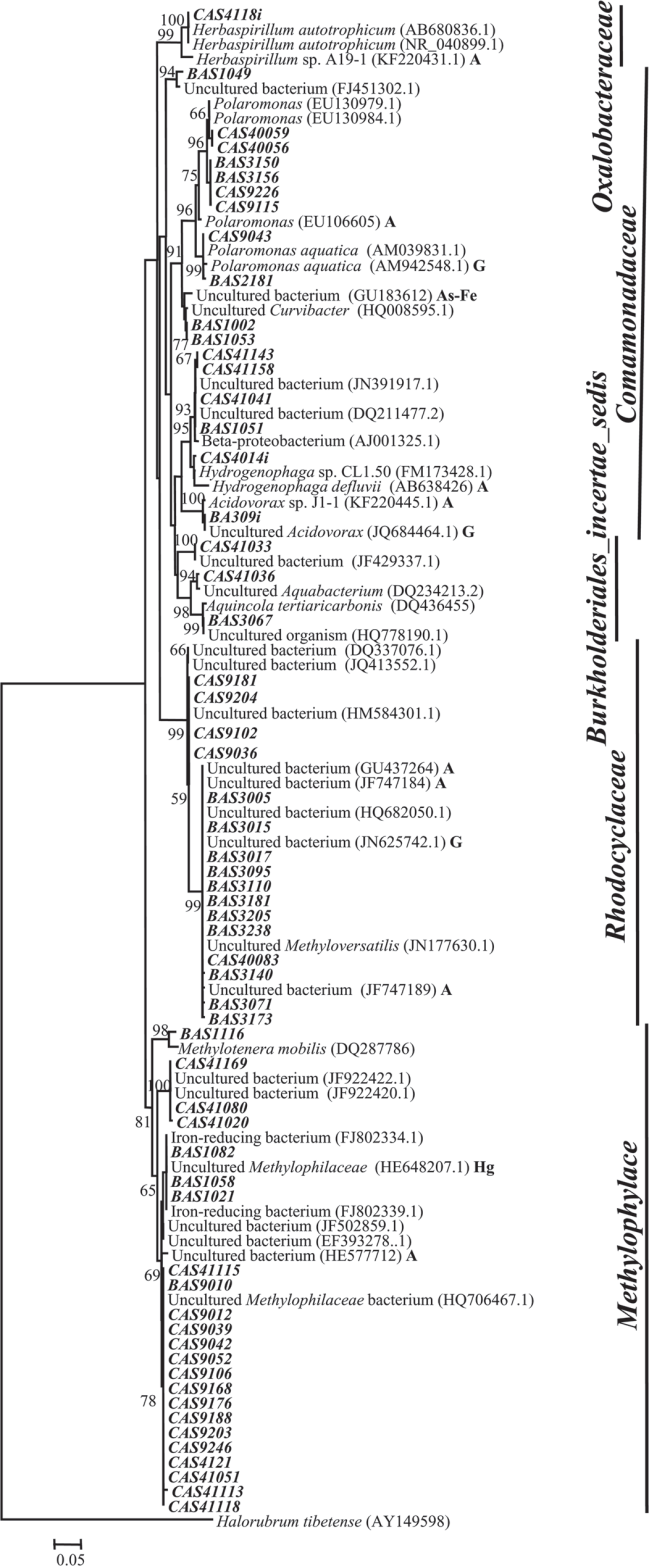
Neighbor-joining phylogenetic tree of *β-Proteobacteria*. The tree is constructed based on 16S rRNA gene sequences by using Jukes-Cantor distances. 1000 bootstraps analyses are conducted and more than 50% are denoted in nodes. Sequences retrieved from As contaminated environment, groundwater and mercury contaminated sites are suffixed here as ‘A’, ‘G’, and ‘Hg’, respectively. Sequences represented in bold italic font are derived from clone libraries and bold italic font with a suffix ‘*i*’ are derived from isolated strains.

**Fig 4:**
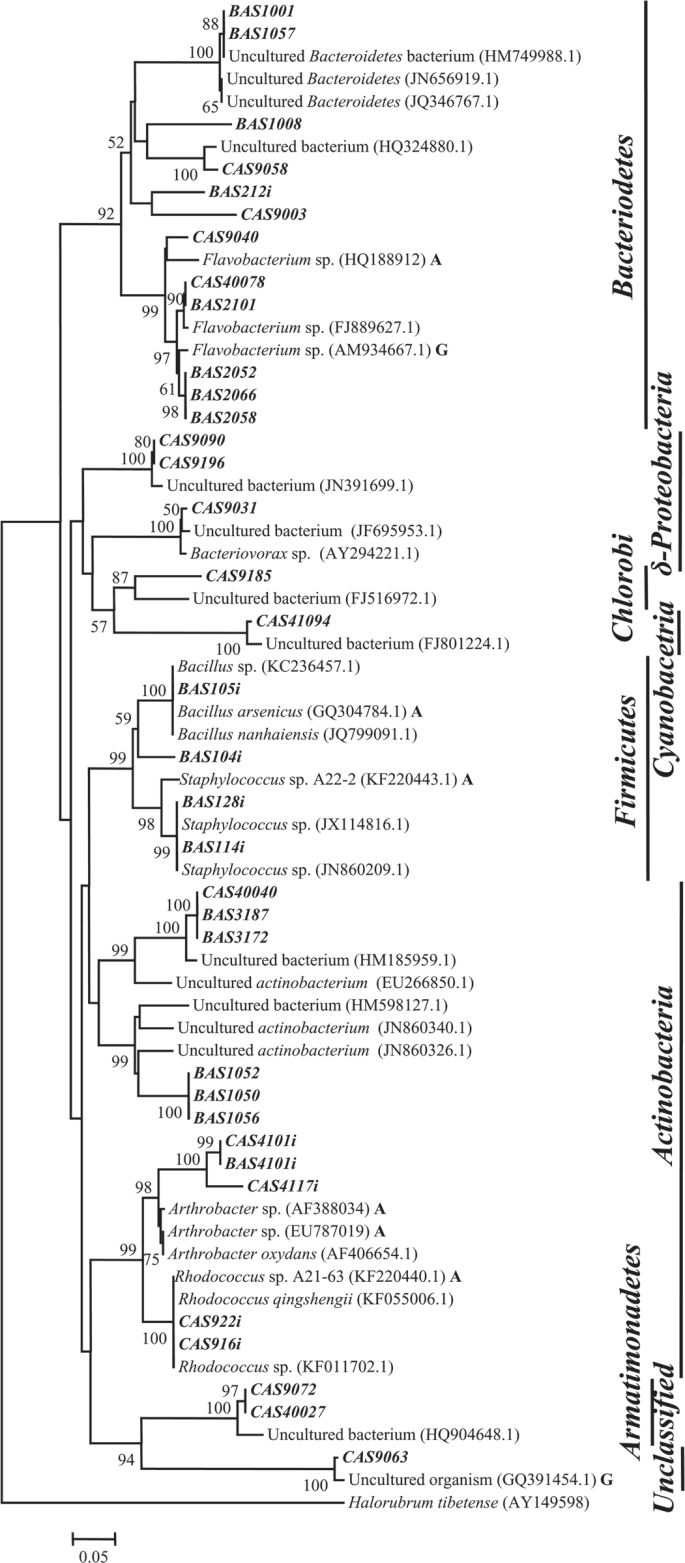
Neighbor-joining phylogenetic tree of *δ-Proteobacteria*, *Actinobacteria*, *Cyanobacteria* and other groups. The tree is based on 16S rRNA gene sequences using by Jukes-Cantor distances. 1000 bootstraps analyses are conducted and more than 50% are denoted in nodes. Sequences retrieved from As contaminated environment and groundwater are suffixed here as ‘A’, and ‘G’, respectively. Sequences represented in bold italic font are derived from clone libraries and bold italic font with a suffix ‘*i*’ are derived from isolated strains.

Members of the genus *Polaromonas* were present in two high as well as two low As samples with maximum abundance of 50% in the high As sample AS40. *Polaromonas* sequences were closely related to *P*. *aquatica* and similar organisms reported from As contaminated water samples. Sequences retrieved from high As samples AS3 and AS9 showed similarity (98%) with *P*. *aquatica* and uncultured *β-Proteobacteria* from As-Fe nodules and formed a separate clade indicating their phylogenetic distinctness (Fig. 3). Methane (or C1 compound) utilizing genera *Methyloversatilis*, *Methylotenera* and *Methylophylaceae* represented the most abundant groups in both high (AS3 and AS9) as well as low As sample (AS41) (Fig. 1B). Sequences affiliated to these groups showed lineage with mostly uncultured bacteria previously retrieved from As contaminated aquifers, As-Fe co-precipitates, Kalahari shield and alkenes’ contaminated river sediment (Fig. 3). Genus *Flavobacterium* was detected as a major population (23%) in low As sample AS2 and as minor group in two other samples (including high As sample AS9) (Fig. 4). Sequences affiliated to this genus showed strong relation with *Flavobacterium* strains isolated earlier from water bodies and As contaminated sediment. Genus *Hydrogenophaga* was detected as moderately abundant population (16%) in both high and low As samples (AS1 and AS41) (Fig. 1B). Sequences of this group showed similarity and lineage with uncultured *β-Proteobacteria* and *H*. *defluvii* reported earlier from As contaminated environment (Fig. 3) [53].

### Less frequently detected bacterial genera

**Fig 5:**
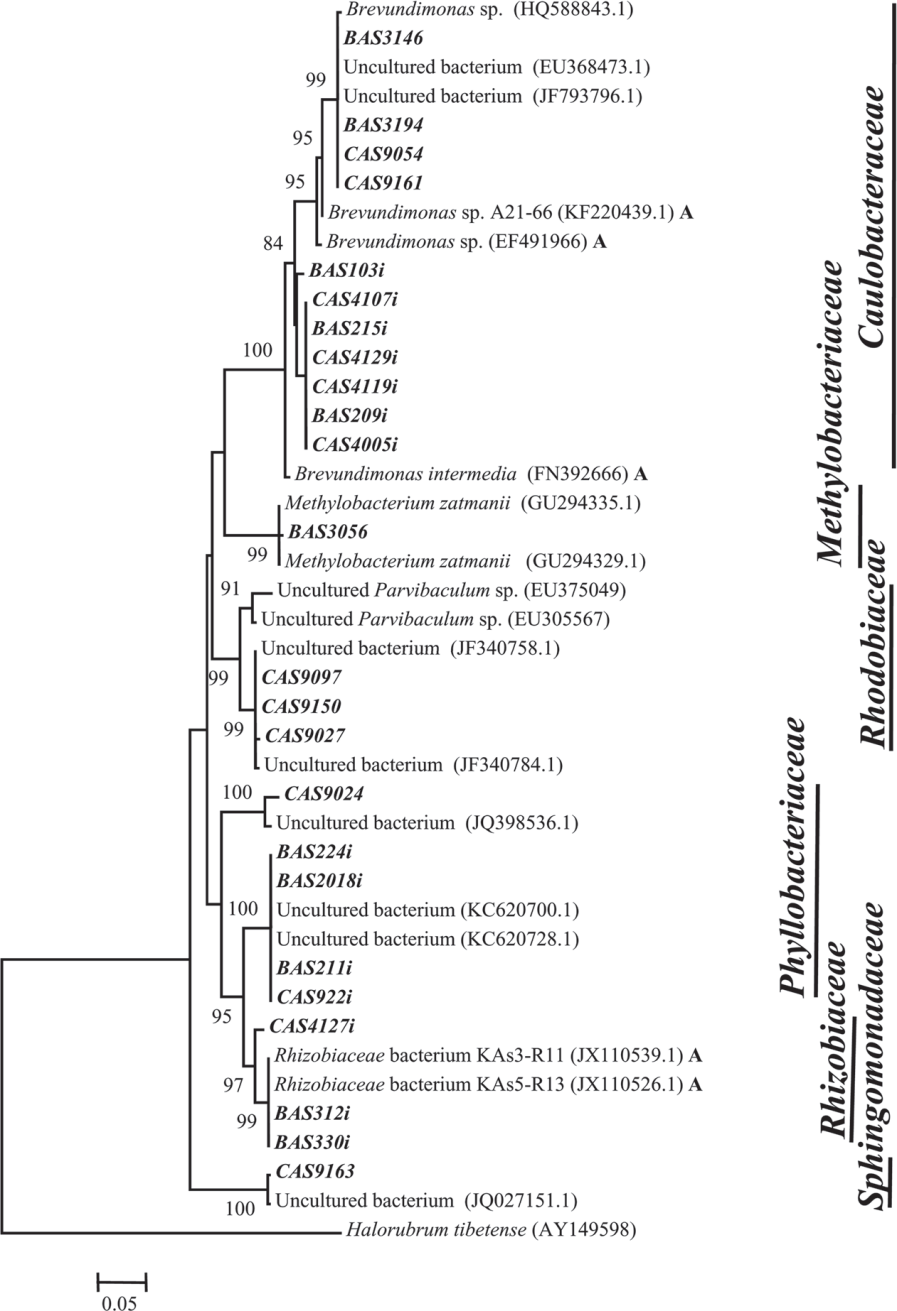
Neighbor-joining phylogenetic tree of α-*Proteobacteria*. The tree is based on 16S rRNA gene sequences by using Jukes-Cantor distances. 1000 bootstraps analyses are conducted and more than 50% are denoted in nodes. Sequences retrieved from As contaminated environment are suffixed as ‘A’. Sequences represented in bold italic font are derived from clone libraries and bold italic font with a suffix ‘*i*’ are derived from isolated strains.

Members of the genera *Aquincola*, *Brevundimonas*, *Parvibaculum*, uncultured *Actinobacteria*, *etc*. of high As samples and genera *Aquabacterium*, *Cyanobacteria* along with unclassified bacteria of low As samples represented the less-frequent and -abundant (<5%) populations. Genera *Rheinheimera* and *Curvibacte*r found in high As sample AS1 as the predominant populations (≥30%) could not be detected elsewhere (Fig. 1B). Genera *Rheinheimera* (*γ-Proteobacteria*) showed close lineage with *Rheinheimera* strains previously reported from various freshwater as well as As contaminated environments (Fig. 2). Sequences related to *Curvibacte*r and *Brevundimonas* showed lineage to similar organisms previously obtained from As contaminated sites including the groundwater from Brahmaputra river basin (Figs. 3, 5).

### PCR-DGGE analysis

Bacterial community structures within the samples were further elucidated using DGGE fingerprinting of PCR amplified 16S rRNA gene. Separation of expected 170–220 bp PCR fragments by DGGE from the samples produced distinct banding patterns (S2 Fig.). Presence of a few common and/or prominent bands across the samples was observed. All together 13 bands from DGGE gel were sequenced and identified (Table 2). Sequences of bands AS3-1, AS2-3, AS41-7 and AS40-12 showed identity with uncultured and cultured *Pseudomonas* sp. retrieved from various environments. Affiliation with uncultured *γ-* and *δ-Proteobacteria* was shown by AS2-4 and AS9-10, respectively. Bands AS1-5, AS41-8 and AS9-9 showed close lineage with *Acinetobacter* sp., *Acidovorax* sp. and uncultured *Methylophilaceae* bacterium, respectively. Sequences of a few bands, *e*.*g*., AS3-2, AS1-6, AS9-11 and AS40-13 showed identity with uncultured unidentified bacteria. It was noted that overall data corresponded well with the clone library based observations.

**Table 2:** Taxonomic affiliation of 16S rRNA gene clones retrieved from major DGGE bands.

Sample ID	Max. identity	NCBI match
AS3-1	99%	*Pseudomonas* sp. (KF557601.1)
AS3-2	100%	Uncultured bacterium clone (KC666823.1)
AS2-3	100%	Uncultured *Pseudomonas* sp. (JQ792937.1)
AS2-4	100%	Uncultured *gamma proteobacterium* (KF379600.1)
AS1-5	98%	*Acinetobacter* sp. (KF551138.1)
AS1-6	100%	Uncultured bacterium isolate DGGE (GQ996402.1)
AS41-7	100%	Uncultured *Pseudomonas* sp. (JF818149.1)
AS41-8	99%	*Acidovorax* sp. (KC914550.1)
AS9-9	98%	Uncultured *Methylophilaceae* bacterium (KF379622.1)
AS9-10	100%	Uncultured *delta proteobacterium* (KF379626.1)
AS9-11	100%	Uncultured bacterium clone (JF696029.1)
AS40-12	100%	*Pseudomonas orientalis* (KF436715.1)
AS40-13	99%	Uncultured bacterium isolate DGGE (GQ996402.1)

### Identification of cultured bacterial isolates

**Fig 6:**
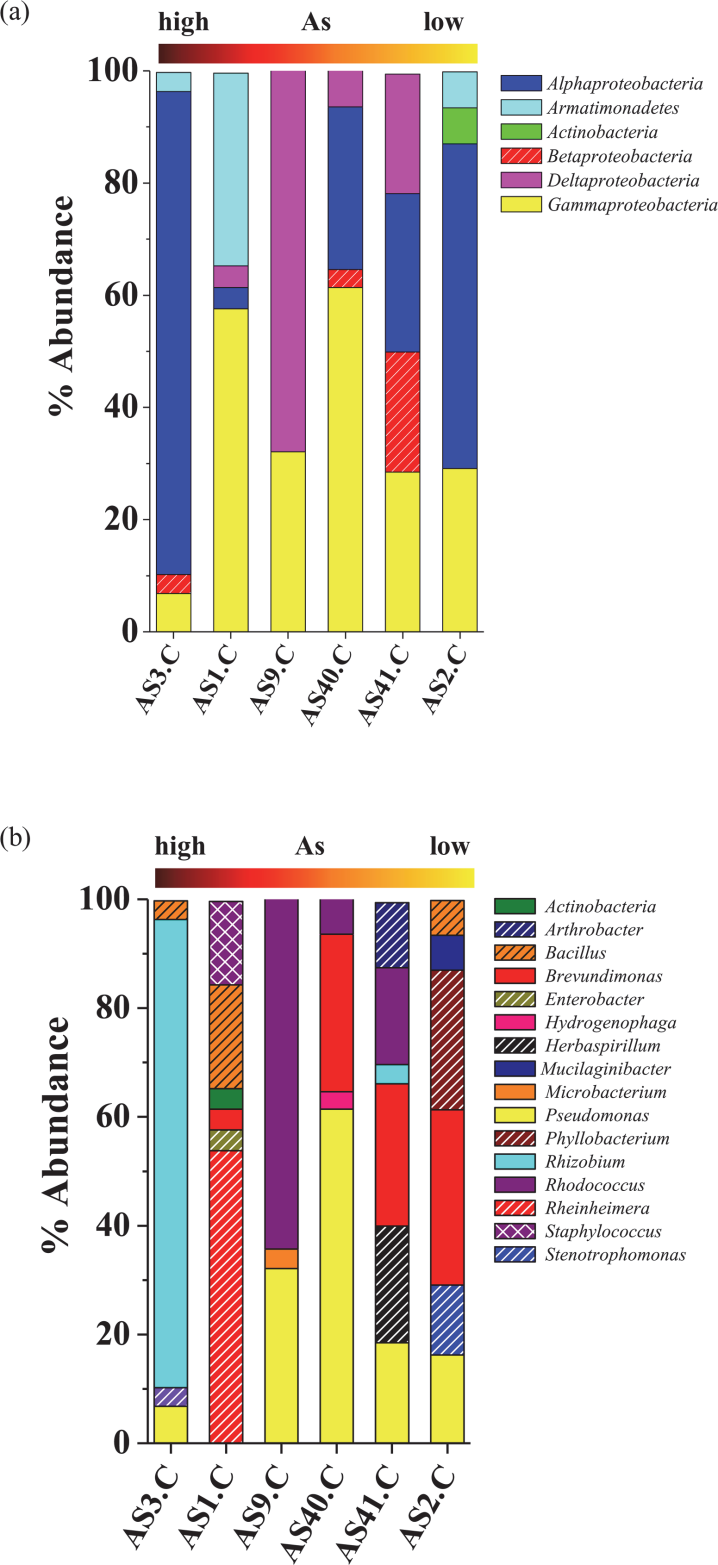
Distribution of major phylogenetic groups of isolated bacteria. Abundance of bacterial groups is plotted with respect to their affiliation at (a) phylum level and (b) genus level. The horizontal bar indicates gradient of As concentrations as detected in six samples.

As many as 173 bacterial strains isolated in the present study were identified and belonged to 16 genera under four phyla (Fig. 6). Isolates affiliated to the genus *Pseudomonas* were most frequently detected (five out of six samples) with moderate to high abundance (7^_^61%). Genera *Brevundimonas*, *Rhodococcus* and *Bacillus* were present in several (≥3) samples, representing major parts of culturable populations. Bacterial strains affiliated to the genera *Rhizobium*, *Rheinheimera*, *Phyllobacterium*, *Herbaspirillum*, *Stenotrophomonas* and *Arthrobacter* though detected less frequently (≤2 samples), they often showed higher abundance (10^_^86%). A few strains were identified as *Hydrogenophaga*, *Microbacterium*, and *Mucilaginibacter* representing minor populations (3^_^6%).

*Pseudomonas* strains retrieved from both high and low As samples showed lineage to several cultured *Pseudomonas* spp. as well as with uncultured *γ-Proteobacteria* previously reported from various subsurface environments (Fig. 2). Genus *Rheinheimera* represented the predominant culturable population (53%) in As rich sample AS1 and its sequences showed relatedness to other *Rheinheimera* sp. and uncultured *γ-Proteobacteria* bacteria retrieved earlier from subsurface or Cr contaminated environment (Fig. 2). Genus *Brevundimonas* was detected as a predominant (24^_^32%) group in three low As samples and as a minor (4%) population in one high As sample (AS1) (Fig. 6B). It was noted that *Brevundimonas* sequences bear strong lineage with uncultured *α-Proteobacteria* bacterium, *B*. *intermedia* and other *Brevundimonas* reported from As contaminated groundwater of Assam (India) and other places (Fig. 5). Genus *Rhodococcus* was found only within the samples from Chakdaha area, of which its abundance was maximum (64%) in As rich sample AS9 (Fig. 6B). Phylogenetic analysis revealed their close lineage with *R*. *qingshengi* and a *Rhodococcus* strain retrieved recently from As contaminated groundwater of Assam (India) (Fig. 4). Genus *Bacillus* was present in both high and low As samples and its sequences showed strong phylogenetic similarity with *B*. *arsenicus* and *B*. *Nanhaiensis* strains recovered earlier from As contaminated environment or sea coast of West India, respectively. Bacterial genera *Staphylococcus* as well as *Arthrobacter* and *Herbaspirillum* were found as abundant groups (≥10%) in AS1 or AS41 sample (Fig. 6B). Sequences of these genera showed close lineages with members of similar groups reported earlier from various As contaminated environments. Genus *Rhizobium* was present in both low and high As samples. Its abundance was higher in As rich sample AS3. Sequences related to this genus showed close lineage to unclassified *Rhizobiaceae* members previously reported from a As contaminated sample of West Bengal [26] (Figs. 5, 6B).

Similar to the clone library based observation, culturable populations also indicated frequent presence of a number of genera (*e*.*g*., *Pseudomonas*, *Brevundimonas* and *Rhodococcus*) across the samples. Members of the genera *Phyllobacterium*, *Herbaspirillum*, *Stenotrophomonas*, *Arthrobacter* and *Hydrogenophaga*, however, could be isolated from only one of the samples. Our results on community composition indicated the complementary nature of culture-independent and -dependent approaches, although, some degrees of overlap with respect to a few taxa were also found. Genera *Pseudomonas*, *Rheinheimera*, *Brevundimonas* and *Hydrogenophaga* were detected by both the approaches, while *Stenotrophomonas*, *Enterobacter*, *Acidovorax*, *Herbaspirillum*, *Phyllobacterium*, *Rhizobium*, *Rhodococcus*, *Microbacterium*, *Bacillus* and *Staphylococcus* could only be isolated, but not found within the 16S rRNA clone library. Genera *Methyloversatilis*, *Methylotenera*, *Polaromonas*, *Curvibaculum* and *Methylobacterium* were only detected through culture-independent approach.

### Arsenic resistance and metabolic characterization of bacterial isolates

Maximum tolerable concentrations (MTC) of As^3+^ and As^5+^ for all the 173 strains was determined following growth with graded concentration of respective As species under aerobic and anaerobic conditions. Prior to the As resistance study, ability of all the test bacteria to grow under anaerobic condition was tested. Nearly 60% of the strains, mostly affiliated to the genera *Acinetobacter*, *Brevundimonas*, *Pseudomonas*, *Rheinheimera*, *Rhizobium*, *Rhodococcus*, *Staphylococcus*, *Stenotrophomonas*, *etc*. were able to grow anaerobically (Table 3). Resistance to As (either As^3+^ or As^5+^ or both) under aerobic as well as anaeorobic condition with considerably high MTC values was found to be an omnipresent phenotype of the bacterial strains. *Actinobacteria*, *Rhizobium* and *Rhodococcus* strains from As rich AS1, AS3, and AS9 samples, respectively showed relatively higher MTC values [≥100 mM] for As^5+^ under both conditions (Table 3). Members of the genera *Pseudomonas*, *Rheinheimera*, *Brevundimonas* and *Herbaspirillum* although showed higher As^5+^ resistance under aerobic condition, their inability to withstand the same under anaerobic condition was noted. Resistance to As^3+^ (MTC ≥5 mM) under aerobic condition was found widely distributed among *Rhizobium* (AS3), *Rhodococcus* and *Pseudomonas* (AS9), and *Bacillus* (AS1 and AS2) as well as in *Acinetobacter* and *Rheinheimera* strains. Under anaerobic condition comparable As^3+^ resistance was mainly found within *Rhizobium* (AS3) and *Rhodococcus* (AS9) strains and within a few *Pseudomonas* strains from AS9 sample.

**Table 3:** Percentile distribution of positive phenotypic test results among the bacterial strains isolated from arsenic contaminated groundwater.

*Genus*	nos	*Origin of strain* ^$^	Phenotypic characteristic																							
			*1*	*2*	*3*	*4*	*5*	*6*	*7*	*8*	*9*	*10*	*11*	*12*	*13*	*14*	*15*	*16*	*17*	*18*	*19*	*20*	*21*	*22*	*23*	*24*
*Acinetobacter*	4	AS1	75	+	75	+	+	25	+	+	+	+	75	+	+	+	+	75	-	50	75	25	25	25	-	25
*Arthrobacter*	4	AS41	33	+	-	+	66	33	+	+	+	+	+	+	+	+	+	+	-	67	33	-	-	33	-	33
*Bacillus*	4	AS1, AS2	60	20	50	75	75	40	60	+	40	40	40	60	60	40	80	40	-	80	60	20	20	20	20	-
*Brevundimonas*	28	AS2, AS41, AS40	52	70	7	74	52	7	81	+	+	+	96	96	+	52	96	+	-	48	44	-	7	18	-	7
*Herbaspirillum*	5	AS41	20	+	-	20	20	-	80	+	+	+	+	+	+	+	+	+	-	20	20	-	40	-	-	20
*Phyllobacterium*	8	AS2	37	25	12	50	50	-	50	75	62	62	62	62	62	62	62	62	-	50	50	25	-	12	-	25
*Pseudomonas*	36	AS2, AS3, AS9, AS41, AS40	46	88	23	63	28	14	68	97	88	94	80	85	88	83	86	88	3	46	34	8	60	8	40	28
*Rheinheimera*	12	AS1	+	91	8	91	25	-	25	+	75	58	75	83	67	50	92	50	-	33	58	17	41	33	8	41
*Rhodococcus*	25	AS9, AS41, AS40	76	92	60	84	76	60	52	96	96	96	96	96	96	96	96	92	20	56	56	-	32	36	20	28
*Staphylococcus*	7	AS1	75	62	25	75	87	12	37	+	75	75	62	75	62	75	87	50	-	50	62	12	25	73	12	50
*Stenotrophomonas*	4	AS2	+	25	-	50	+	-	50	+	50	50	50	50	50	25	50	50	-	25	-	-	-	-	-	25
*Rhizobium*	33	AS3, AS41, AS40	54	93	69	93	66	-	48	93	78	84	87	87	84	69	84	84	51	69	18	33	30	39	3	21
*Hydrogenophaga*	1	AS40	-	+	-	+	-	-	-	+	+	+	+	+	+	+	+	+	-	+	+	-	-	-	-	-
*Microbacterium*	1	AS9	+	+	+	+	-	+	+	+	+	+	+	+	+	+	+	+	-	-	-	-	-	-	-	-
*Mucilaginibacter*	1	AS2	-	-	-	-	-	-	-	+	+	+	+	-	-	-	-	-	-	-	-	-	-	-	-	-

Phenotypic characteristics are 1: anaerobic growth; 2: As^5+^ aerobic tolerance; 3: As^5+^ anaerobic tolerance; 4: As^3+^ aerobic tolerance; 5: As^3+^ anaerobic tolerance; 6: arsenite oxidase; 7: arsenate reductase; 8: casein; 9: glycerol; 10: glucose; 11: acetate; 12: pyruvate; 13: lactate; 14: citrate; 15: starch; 16: sucrose; 17: ascorbic acid; 18: As^5+^; 19: Si^6+^; 20: SO_4_^2−^; 21: S_2_O_3_^2−^; 22: SO_3_^2−^; 23: NO_3_^−^; 24: Fe^3+^. ‘+’: 100% positive; ‘–’: 100% negative, nos: number of bacterial strains affiliated to particular genus as used in the present analysis, $: Sample ID from which particular strains are isolates

**Fig 7:**
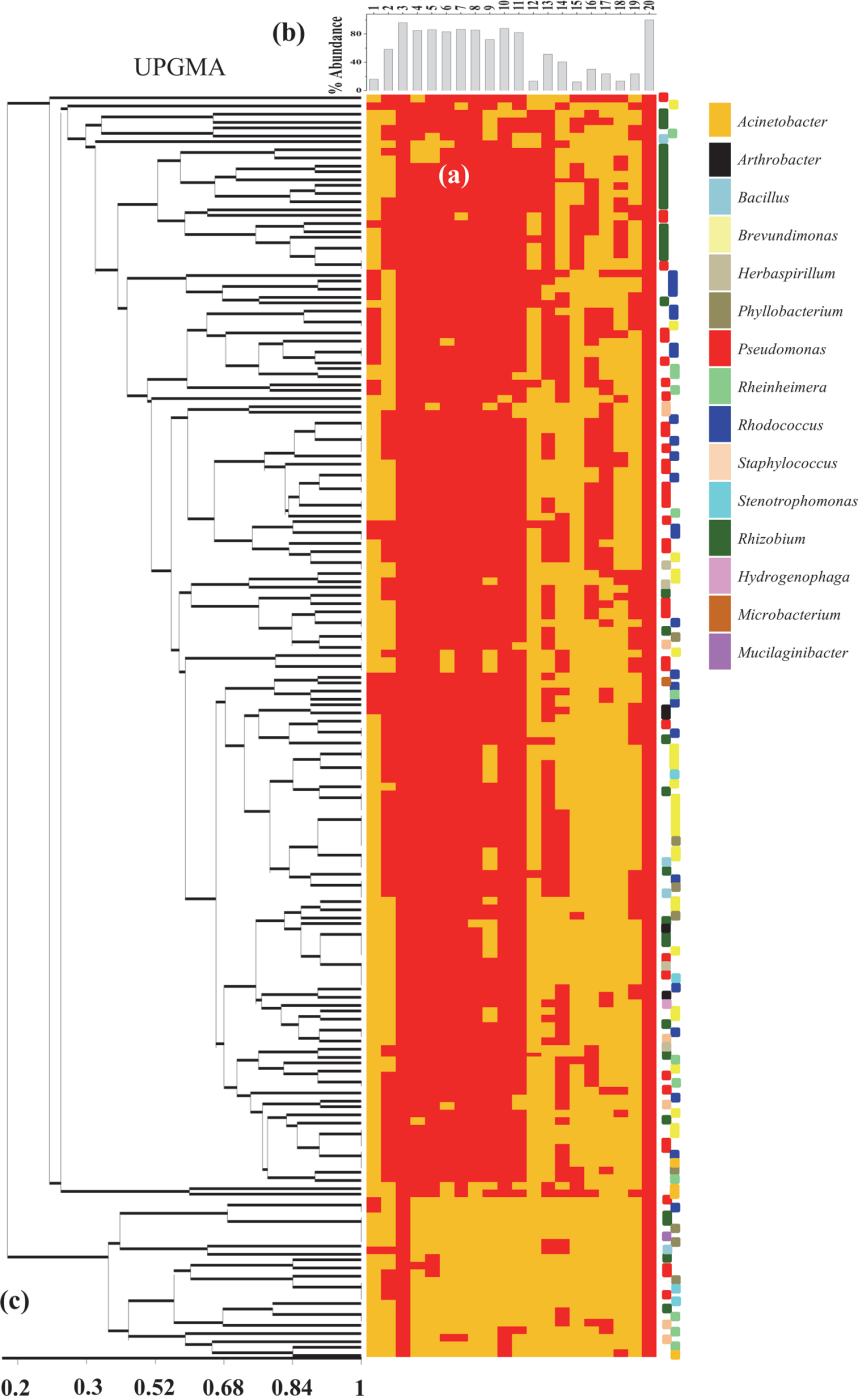
Consolidated profiles of all the metabolic properties tested within the bacterial strains isolated from six As contaminated samples. (a) Positive (red box) and negative (yellow box) responses obtained for various properties tested; (b) Percentage of positive response obtained for each property and (c) UPGMA based on metabolic properties of bacterial strains isolated from six As contaminated samples. Metabolic characters tested are (I) enzymes involved in As transformation: 1: arsenite oxidase; 2: arsenate reductase; (II) ability to utilize different carbon sources: 3: cacsin; 4: glycerol; 5: glucose; 6: acetate; 7: pyruvate; 8: lactate; 9: citrate; 10: starch; 11: sucrose; 12: ascorbic acid and (III) utilization of different terminal electron acceptors: 13: As^5+^; 14: Se^6+^; 15: SO_4_^2−^; 16: S_2_O_3_^2−^; 17: SO_3_^2−^; 18: NO_3_^−^; 19: Fe^3+^; 20: O_2._ Taxonomic affiliations of bacterial isolates are indicated using coloured bars presented right side of consolidated metabolic profiles.

Metabolic characteristics of bacterial populations were ascertained by a number of tests including As^3+^ oxidase and As^5+^ reductase activities, utilization potential of several carbohydrates and different inorganic electron acceptors (Fig. 7). A consolidated profile of all the metabolic properties tested along with percentage of positive response obtained for each trait was presented in Fig. 7A. Arsenate reductase activity was found within nearly two third of the bacterial strains mostly affiliated to genera *Brevundimonas* (from samples AS2, AS40 and AS41), *Pseudomonas* (AS2, AS9, AS40 and AS41), *Rhodococcus* (AS9), *Rhizobium* (AS3), *Phyllobacterium* (AS2), *Acinetobacter (*AS1) and *Arthrobacter* (AS41) (Table 3). In comparison to this, As^3+^ oxidase activity was less abundant (16%), present within the strains mostly affiliated to genera *Rhodococcus* and *Pseudomonas* from AS9 sample. With respect to utilization of different carbon/electron sources, more than 80% of the isolates were able to metabolize all the compounds tested, except citrate and ascorbate. The latter two compounds were metabolized by 72% and 13% of the isolates, respectively (Fig. 7B). Interestingly, members of a few genera like *Bacillus*, *Phyllobacterium*, *Staphylococcus* and *Stenotrophomonas* showed their inability to utilize many of the test compounds (*e*.*g*., glucose, glycerol, acetate, pyruvate, *etc*.). With respect to utilization of different inorganic elements as terminal electron acceptors (TEAs) during anaerobic growth, 50% of the strains showed their ability to metabolize As^5+^ followed by Se^6+^ (40%), S_2_O_3_^2−^ (30%), Fe^3+^ and HSO_4_^−^ (24%), NO_3_^−^ (13%) and SO_4_^2−^ (12%) (Fig. 7B). Ability to grow anaerobically using As^5+^ as TEA was found to be relatively frequent within a few genera isolated from specific samples (*e*.*g*., *Rhizobium* strains from AS3, *Rhodococcus* strains from AS9 and *Bacillus* strains from AS2) (Table 3). Ability to use Fe^3+^ as TEA was mainly present in *Rhodococcus*, *Pseudomonas* and *Brevundimonas* strains from AS9, AS40 and AS41 along with a few *Rhizobium* (AS3), *Staphylococcus* (AS1) and *Phyllobacterium* (AS2) strains. Interrelation among the test metabolic properties indicated presence of several distinct groups across the taxonomic domains (Fig. 7C). The UPGMA dendrogram clearly showed that bacterial strains belonged to taxonomically distinct groups share metabolic traits at high similarity (> 80%).

**Fig 8:**
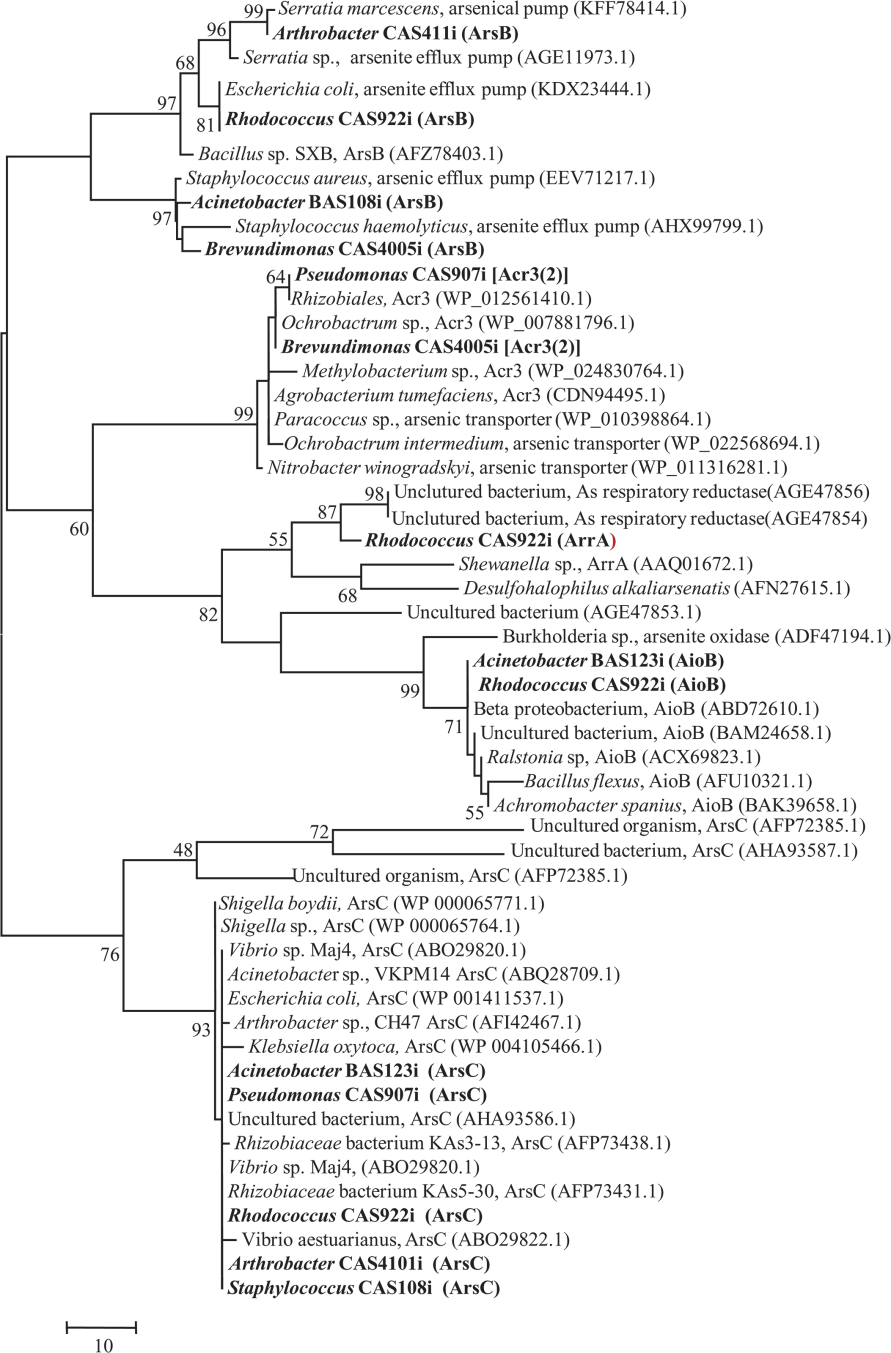
Phylogenetic tree of deduced amino acid sequences of genes encoding As^5+^ reductase (*ars*C and *arr*A), As^3+^ oxidase (*aio*B), and As^3+^ efflux [*ars*B, and *acr*3(2)]. Genes detected in this study are shown in bold font. Percentage values on each branch represent the corresponding bootstrap probability values obtained in 500 replications. Significant bootstrap values (>50%) of major branch points are shown in the tree.

**Fig 9:**
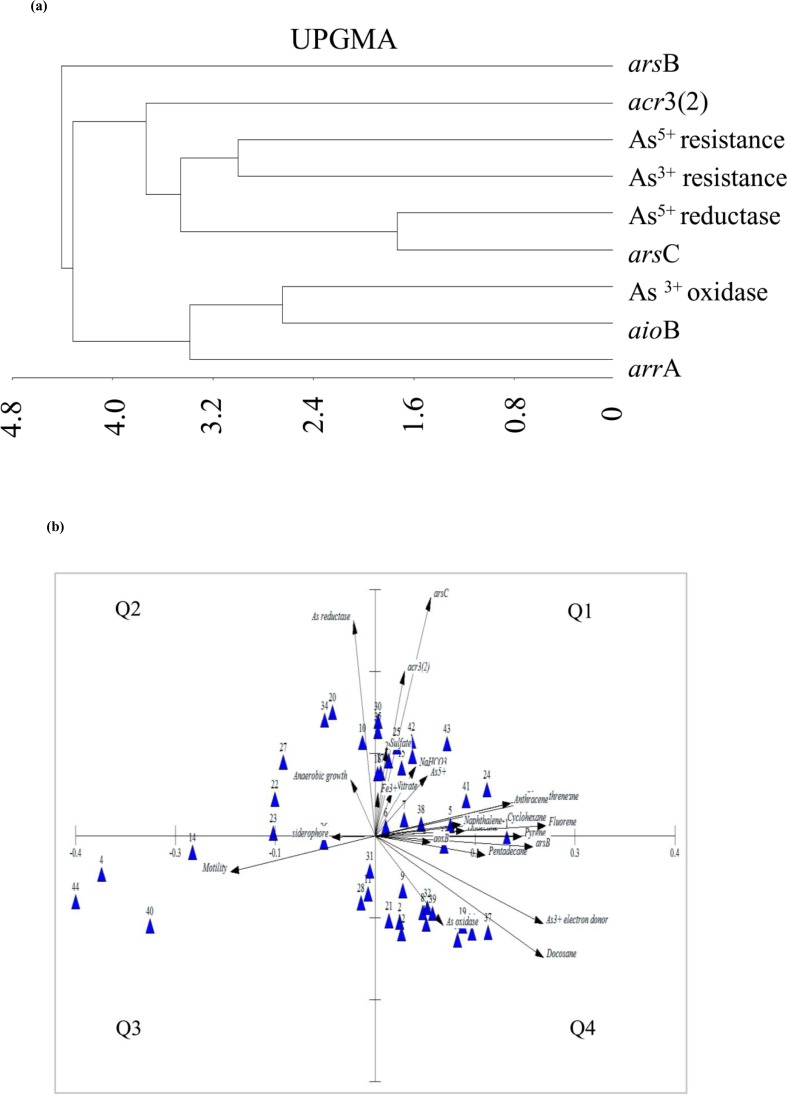
Correlation among the various metabolic properties and As related genes detected within selected 44 isolates. (a) UPGMA cluster analysis based on selected genetic and metabolic characters (*viz*., As related genes, As-resistance and -transformation abilities) and (b) plot of PCA scores on utilization of different C sources and alternate terminal electron acceptors, As^3+^ oxidase and As^5+^ reductase activities, As resistance genes, motility and siderophore production of the isolates. In 9(b), each bacterial strain is represented by numbers referring to their names (S5 Table); arrows indicate corresponding parameters for which the isolates with higher eigen values have clustered.

Along with these properties, presence of genes responsible for As transformation (*ars*C, *arr*A and *aio*B) and efflux [*ars*B and *acr*3(2)], utilization of As^3+^ as electron donor and HCO_3_^−^ or long chain aliphatic and polyaromatic hydrocarbons as carbon source, production of siderophore and motility were also studied within selected 44 strains (Table 4). These strains were selected based on their taxonomic abundance and members of nearly all genera were included. Nearly 50% of the test strains belonged to the genera *Pseudomonas*, *Rhizobium*, *Brevundimonas*, *Rhodococcus*, *etc*., showed presence of cytosolic As^5+^ reductase *ars*C gene. Sequences of *arsC* gene obtained from *Acinetobacter* BAS123i, *Arthrobacter* CAS410i, *Pseudomonas* CAS907i, *Rhodococcus* CAS922i and *Staphylococcus* CAS108i shared high identity and lineages with As^5+^ reductase of several *Proteobacteria* (*Vibrio* sp., *Escherichia coli*, *Acinetobacter* sp., *Ochrobactrum* sp., *Pseudomonas*, *etc*.) (Fig. 8). Arsenate transporter gene (*ars*B) was detected in 24 strains affiliated mostly to the genera *Pseudomonas*, *Brevundimonas*, *Rhodococcus*, *Staphylococcus*, *etc*. Sequences of *ars*B gene obtained from *Brevundimonas* CAS4005i, *Acinetobacter* BAS108i, *Rhodococcus* CAS922i and *Arthrobacter* CAS411i showed relatedness with putative As^3+^ efflux pump from *Serratia*, *Staphylococcus*, *etc*. (Fig. 8). Arsenite transporter gene *acr*3(2) was detected in 14 strains belonging to genera *Pseudomonas*, *Staphylococcus*, *Arthrobacter*, *Rhizobium*, *etc*. Sequences of *acr*3(2) gene detected in these strains showed lineages with As^3+^ transporters/efflux pumps reported from genera *Ochrobactrum*, *Rhizobium*, *etc*. (Fig. 8). The *aio*B gene encoding As^3+^ oxidase was present in five bacterial strains affiliated to genera *Rhodococcus*, *Acinetobacter*, *Staphylococcus* and *Microbacterium*. Nucleotide sequences of this gene showed their closeness with As^3+^ oxidase of *Bacillus*, *Achromobacter* and with several uncultured *β*- and *α*-proteobacterial members (Fig. 8). Dissimilatory As^5+^ reductase gene *arr*A was relatively less abundant and could be amplified successfully from only seven strains affiliated to genera *Rhodococcus*, *Pseudomonas* and *Brevundimonas*. The sequence obtained from *Rhodococcus* CAS922i revealed its close lineage with *arr*A of *Shewanella* sp. Noticeably, a number of strains showed simultaneous presence of *ars*C, *ars*B and *acr*3(2) or *ars*C and *arr*A genes. Possible relation among the presence of all these genes detected within the bacterial strains and their As-resistance and -transformation phenotypes was studied by UPGMA (Fig. 9A). Presence of *ars*C gene was found to be strongly related with As^5+^ reductase activity and together with *acr*3(2) gene, a close agreement between As resistance, transformation and presence of both these As related genes was evident.

**Table 4:** Details of the results obtained for As^3+^ and As^5+^ resistance, utilization of different hydrocarbon compounds and HCO_3_ as carbon source, As^3+^ as electron donor, siderophore production, motility and presences of As related genes within selected strains isolated from six As contaminated groundwater samples.

*Strains*			*Biochemical tests*														*As related genes*				
	As^3+^ resistance	As^5+^ resistance	Nonadecane	Docosane	Dodecane	Pentadecane	Cyclohexane	Phenanthrene	Naphthalene	Pyrene	Fluorene	Anthracene	NaHCO_3_	As^3+^ e donor	Motility	Siderophore	*ars*B	*ars*C	*acr*3(2)	*aio*B	*arr*A
*Acinetobacter BAS123i	+	+	+	+	+	+	+	+	+	+	+	+	+	+	-	-	+	+	-	+	-
*Arthrobacter* CAS4117i	+	+	+	+	+	+	+	+	+	+	+	+	+	+	+	-	-	-	-	-	-
*Arthrobacter CAS4101i	+	+	+	-	+	+	+	+	+	+	+	+	+	+	+	+	+	+	+	-	-
*Bacillus* BAS204i	-	-	-	-	-	-	-	-	-	-	-	-	+	-	+	-	-	-	-	-	-
*Brevundimonas* BAS230i	-	-	+	+	+	+	+	+	+	+	+	+	+	+	+	-	+	+	-	-	-
*Brevundimonas* CAS4119i	-	-	+	+	+	+	+	+	+	+	+	+	+	-	+	-	-	+	-	-	-
*Brevundimonas* CAS4123i	-	+	+	+	+	+	+	+	+	+	+	+	+	-	+	-	+	+	-	-	-
*Brevundimonas* CAS4008i	+	-	+	+	+	+	+	+	+	+	+	+	+	-	+	-	+	-	-	-	-
*Brevundimonas* BAS223i	+	-	+	+	+	+	+	+	+	+	+	+	+	-	+	-	+	-	-	-	-
*Brevundimonas CAS4005i	+	+	+	-	+	-	+	+	+	+	+	+	+	-	+	-	+	+	+	-	+
*Herbaspirillum* CAS4110i	-	-	+	+	+	+	+	+	+	+	+	+	+	-	+	-	-	-	-	-	-
*Hydrogenophaga* CAS4014i	+	+	+	+	+	+	+	+	+	+	+	+	-	+	+	-	-	-	-	-	-
*Microbacterium* CAS905i	+	+	+	+	-	+	+	+	+	+	+	+	-	+	+	-	+	-	-	+	-
*Phyllobacterium* BAS211i	+	-	-	-	+	+	-	-	+	+	-	+	-	-	+	-	-	-	-	-	-
*Phyllobacterium BAS224i	-	-	+	-	+	+	+	+	+	+	+	+	+	-	-	-	+	+	-	-	-
*Pseudomonas* CAS934i	+	+	+	+	+	+	+	+	+	+	+	+	+	+	+	-	+	-	-	-	-
*Pseudomonas* CAS4116i	+	-	+	+	+	+	+	+	+	+	+	+	+	-	+	-	-	+	+	-	-
*Pseudomonas* CAS4106i	-	-	+	+	+	+	+	+	+	+	+	+	+	-	+	-	-	+	+	-	+
*Pseudomonas* CAS4105i	-	-	+	+	+	+	+	+	+	+	+	+	+	+	-	-	+	-	-	-	-
*Pseudomonas* BAS309i	-	-	+	-	+	-	+	+	+	+	+	+	+	-	+	-	-	+	+	-	-
*Pseudomonas* CAS4016i	+	+	+	+	+	+	+	+	+	+	+	+	-	-	+	-	+	-	-	-	-
*Pseudomonas* CAS908i	+	+	+	-	+	+	-	+	+	-	-	+	+	-	+	-	+	+	-	-	+
*Pseudomonas* CAS4001i	+	+	-	-	+	-	+	+	+	+	+	-	+	-	+	+	+	-	-	-	+
*Pseudomonas BAS323i	+	+	+	+	+	+	+	+	+	+	+	+	+	+	-	-	+	+	-	-	-
*Pseudomonas CAS907i	+	+	+	-	-	+	+	+	+	-	+	+	+	+	-	-	+	+	+	-	+
*Rheinheimera* BAS124i	-	+	+	-	+	+	+	+	+	+	+	+	+	-	+	-	-	-	-	-	-
*Rheinheimera* BAS122i	+	-	+	-	-	+	+	+	+	-	+	+	+	-	+	-	-	+	-	-	-
*Rheinheimera* BAS127i	-	+	+	+	+	+	+	+	+	+	+	+	-	-	+	-	-	-	-	-	-
*Rhizobium* CAS325i	+	+	+	+	+	+	+	+	+	+	+	+	+	-	+	-	-	+	+	-	-
*Rhizobium* BAS306i	+	+	+	-	+	+	+	+	+	+	+	+	-	-	+	-	+	+	+	-	-
*Rhizobium* CAS4026i	+	+	+	-	+	+	+	+	+	+	+	+	+	+	+	-	-	-	+	-	-
*Rhizobium* CAS4022i	+	+	+	+	+	+	+	+	+	+	+	+	+	-	-	-	+	-	-	-	-
*Rhizobium* BAS310i	-	+	+	+	+	+	+	+	+	+	+	+	+	+	-	-	-	-	+	-	-
*Rhizobium* BAS316i	-	-	+	-	+	+	+	+	-	+	-	+	+	-	-	-	-	+	+	-	-
*Rhizobium* BAS305i	-	-	+	-	+	+	+	+	+	+	+	+	+	-	-	-	-	+	-	-	-
*Rhodococcus* CAS912i	+	-	+	+	+	+	+	+	+	+	+	+	+	+	-	-	+	-	-	-	-
*Rhodococcus* CAS931i	+	+	+	+	+	+	+	+	+	+	+	+	+	+	-	-	+	-	-	-	-
*Rhodococcus* CAS930i	+	+	+	-	+	+	+	+	+	+	+	+	+	+	-	-	-	-	+	-	+
*Rhodococcus* CAS933i	+	+	+	-	+	+	+	+	-	+	+	+	-	+	-	-	+	-	-	+	-
*Rhodococcus* CAS4021i	+	+	-	-	+	+	+	-	+	-	-	-	-	-	+	+	-	-	-	-	-
*Rhodococcus CAS922i	+	+	+	-	+	+	+	+	+	+	+	+	+	+	-	-	+	+	-	+	+
*Staphylococcus* CAS106i	+	+	+	-	+	+	+	+	+	+	+	+	+	-	-	-	+	+	+	+	-
*Staphylococcus BAS108i	+	+	+	+	+	+	+	+	+	+	+	+	+	-	-	-	+	+	+	-	-
*Stenotrophomonas* BAS202i	-	-	-	-	-	-	-	-	-	-	-	-	+	-	+	-	-	-	-	-	-

Note: For As^3+^ and As^5+^ resistance, + ve sign indicates maximum tolerable concentration ≥ 5mM and ≥ 50mM, respectively. Bacterial strains with bold font are used in microcosm study.

Ability to grow autotrophically using HCO_3_^−^ or heterotrophically utilizing a range of long chain aliphatic and polyaromatic hydrocarbons was found to be ubiquitous within the bacterial strains. Comparatively, a fewer strains showed their ability to use As^3+^ as electron donor. In particular, *Rhodococcus* strains from AS9, *Arthrobacter* strains from AS41 and a few *Pseudomonas*, *Rhizobium* strains were capable of chemolithotrophic metabolism using As^3+^. Among the test hydrocarbons, except docosane rest all were metabolized by most (>86%) of the strains. Motility was detected within 63% of the bacterial isolates. Ability to produce siderophore was scarce and three isolates namely, *Arthrobacter* CAS4101i and CAS4117i and *Rhodococcus* CAS4021i were found positive.

Interrelationship among the various metabolic properties tested within the bacterial strains was further studied by euclidean PCA (Fig. 9B, S5 Table). Based on their metabolic profiles, bacterial strains found to be grouped into distinct clusters and distributed within four quadrants (Fig. 9B). Quadrant 1 and 4 accommodated maximum strains and properties. Within quadrant 1 bacterial strains affiliated to genera *Pseudomonas*, *Rhizobium*, *Rhodococcus*, *Brevundimonas* and *Staphylococcus* showed higher level of agreement with respect to presence of *ars*C and *acr*3(2) genes, utilization of As^5+^, Fe^3+^, NO_3_^−^ and SO_4_^2−^ as TEA and use of acetate, lactate, starch, anthracene, naphthalene cyclohexane and bicarbonate, as carbon source (Fig. 9B). On the other hand, As^3+^ oxidase activity, *aio*B gene, utilization of As^3+^ as electron donor and docosane as carbon source by a few *Pseudomonas*, *Rhodococcus*, *Arthrobacter* and *Brevundimonas* strains represented the fourth quadrant (Q4).

### Microcosms study

**Fig 10:**
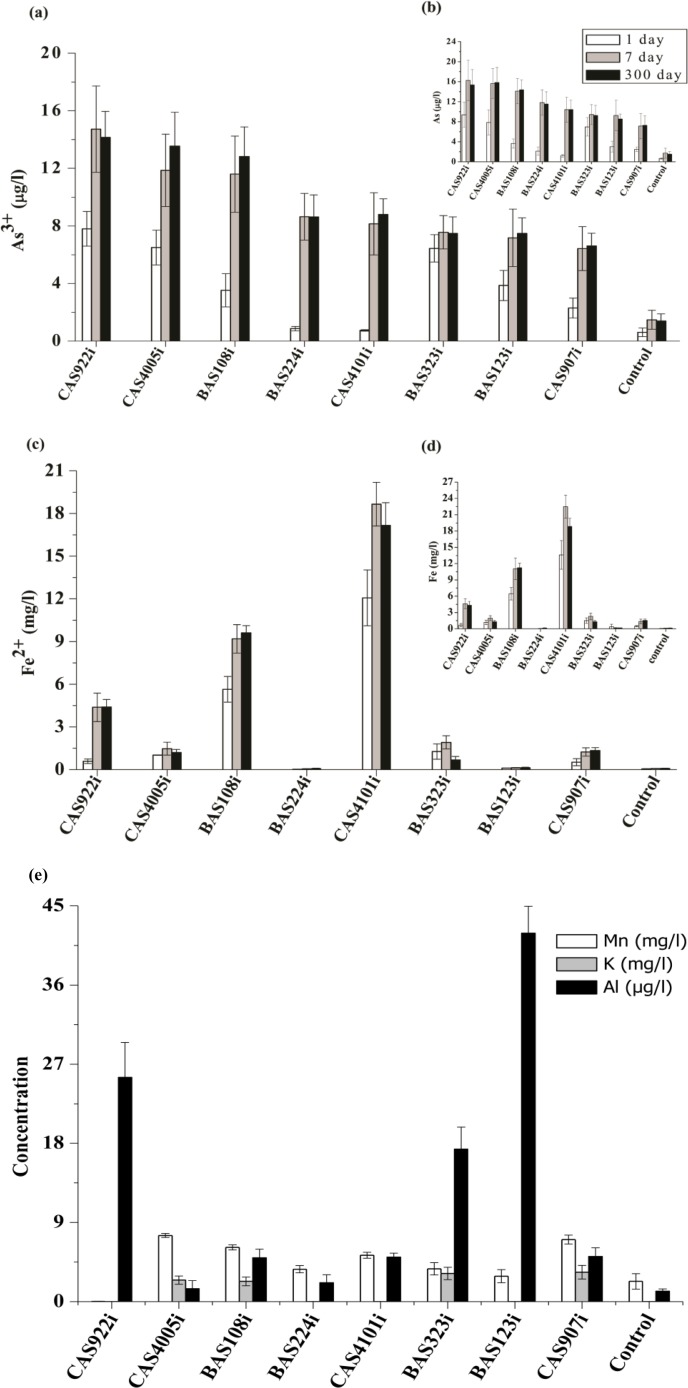
Concentrations of As (a and b), Fe (c and d) and other metals (Mn, K and Al) (e) detected within the aqueous phase of microcosms (after 300 days). Error bars indicate standard deviations (n = 3). Bacterial strains represented in X axis are as follows: CAS922i (*Rhodococcus* sp.), CAS4005i (*Brevundimonas* sp.), BAS108i (*Staphylococcus* sp.), BAS224i (*Phyllobacterium* sp.), CAS4101i (*Arthrobacter* sp.), BAS323i (*Pseudomonas* sp.), BAS123i (*Acinetobacter* sp.) and CAS907i (*Pseudomonas* sp.).

Further to investigate the role of inhabitant bacteria in As mobilization, eight strains were used in sediment microcosm study (Table 4). Considering the metabolic properties and taxonomic affiliation of predominant populations representative strains were selected. All these strains were endowed with As reductase activity, *ars*C, *ars*B and/or *acr*3(2) genes. *Rhodococcus* CAS922i, *Brevundimonas* CAS4005i and *Pseudomonas* CAS907i contain *arr*A gene as well. All the strains except *Pseudomonas* CAS907i and *Acinetobacter* BAS123i could use As^5+^ as TEA while *Staphylococcus* BAS108i and *Arthrobacter* CAS4101i have additional ability to utilize Fe^3+^ as TEA. Biotic microcosms showed that all the bacterial strains could maintain their viability during incubation (up to 300 days) with orange sand and cause As release (S6 Table). Concentration of released As varied with respect to the bacterial strain used; slightly higher As mobilization (>14 μg/l) was noted for *Rhodococcus* CAS922i, *Brevundimonas* CAS4005i and *Staphylococcus* BAS108i strains (Fig. 10). Noticeably, in each biotic microcosm, aqueous As concentration reached to its maximum within the first 7 days and 80^_^90% of released As was found to be in the form of As^3+^. It was noted that strains with *arr*A gene and ability for anaerobic growth utilizing As^5+^ as TEA (*Rhodococcus* CAS922i and *Brevundimonas* CAS4005i) could mobilize relatively higher concentrations of As. In contrast, *Pseudomonas* CAS907i, that showed presence of *arr*A gene but unable to use As^5+^ during anaerobic growth showed release of lesser concentration of As.

Coupled with As, release of Fe [predominantly (90^_^95%) in the form of Fe^2+^] was observed in all the biotic microcosms, however, pattern of Fe and As release did not correlate well among the isolates. During the initial days (1^_^7 days) of incubation, it was noted that Fe reducing strains *Arthrobacter* CAS4101i and *Staphylococcus* BAS108i could lead to release of significantly higher amount of Fe over the other sets and this occurred before the appearance of significant amount of As^3+^ in the aqueous phase. In contrast to this, microcosms with other As^5+^ reducing strains (not capable of Fe^3+^ reduction) *Rhodococcus* CAS922i and *Brevundimonas* CAS4005i showed appearance of As^3+^ followed by Fe^3+^. Compared to all the biotic microcosms, abiotic control sets showed negligible release of As (1.9 μg/l) and Fe (0.08 mg/l) even upto the end of incubation period (300 days) (Fig. 10).

Together with As and Fe, release of other trace elements (Al, Mn, K, *etc*.) was also observed in case of the biotic microcosms. Analyses of both solid and aqueous phases indicated mobilization of several elements through possible dissolution of host minerals of orange sand (Fig. 10E, S7 Table). Release of Al was observed in microcosms with strain *Acinetobacter* BAS123i, *Rhodococcus* CAS922i and *Pseudomonas* BAS323i. Release of Mn (1.4^_^5.2 mg/l) and K (2.7^_^3.8 mg/l) was found in microcosms with *Brevundimonas* CAS4005i, *Staphylococcus* BAS108i, *Pseudomonas* CAS907i and *Pseudomonas* BAS323i. X-ray fluorescence (XRF) analysis of sediment before and after incubation with bacteria corroborated well with the elemental analysis data of aqueous samples indicating reduced abundance of Fe, Al, K, Mn, *etc*. following bacterial incubation (S7 Table). Although release of As was not strongly (S8 Table) correlated (*p≤*0.05) with that of other trace elements tested, nevertheless, in all higher As releasing microcosms mobilization of one or more constituting elements of the sand was obviously noted. Mineralogical analysis confirmed that the orange sand was composed of several As bearing minerals like Fe oxyhydroxide based goethite [FeO(OH)], albite (NaAlSi_3_O_8_) and glauconite (K[(Fe,Al)_2_(Si,Al)_4_O_10_(OH)_2_]). XRD data indicated that following incubation with bacteria there was distinct mineralogical changes (Table 5).

**Table 5:** Mineralogical composition (as determined by XRD analysis) of orange sand before and after 300 days incubation with selected bacterial strains under anaerobic condition.

Minerals		Sample ID									
	Original sediment		Control	BAS108i	BAS123i	BAS323i	CAS907i	CAS922i	CAS4005i	CAS4101i	BAS224i
Muscovite	++++	**After 300 days incubation**	++++	++++	++++	++++	++++	++++	++++	++++	++++
Quartz	++++		++++	++++	++++	++++	++++	++++	++++	++++	++++
Clinochlore	++++		++++	++++	++++	++++	++++	++++	++++	++++	++++
Goethite	++++		++++	+++	++++	+++	+	+++	++++	++	++++
Glauconite	++++		++++	+	+++	++	++	+	+	+	++
Albite	++++		++++	++	+	+	++	+	+++	++	++

The isolates are denoted as follows: CAS922i (*Rhodococcus* sp.), CAS4005i (*Brevundimonas* sp.), BAS108i (*Staphylococcus* sp.), BAS224i (*Phyllobacterium* sp.), CAS4101i (*Arthrobacter* sp.), BAS323i (*Pseudomonas* sp.), BAS123i (*Acinetobacter* sp.) and CAS907i (*Pseudomonas* sp.). (++++: abundant; +++: common; ++: a little amount; +: very less amount).

Since mineral dissolution is often caused by microbially produced organic acids, concentrations of such acids were further estimated within the aqueous phase of microcosms. It was found that oxalic acid is the only organic acid that could be detected at significantly higher concentration (*p≤*0.05) from the aqueous part of microcosms by IC analysis. Although several strains could produce this acid (115^_^562 mg/l), *Brevundimonas* BAS4005i yielded maximum concentration (S3 Fig.). Low concentration of oxalic acid was also detected in abiotic control which could probably be resulted from abiotic transformation of glucose in presence of minerals as also reported earlier by Frey et al. [50].

**Fig 11:**
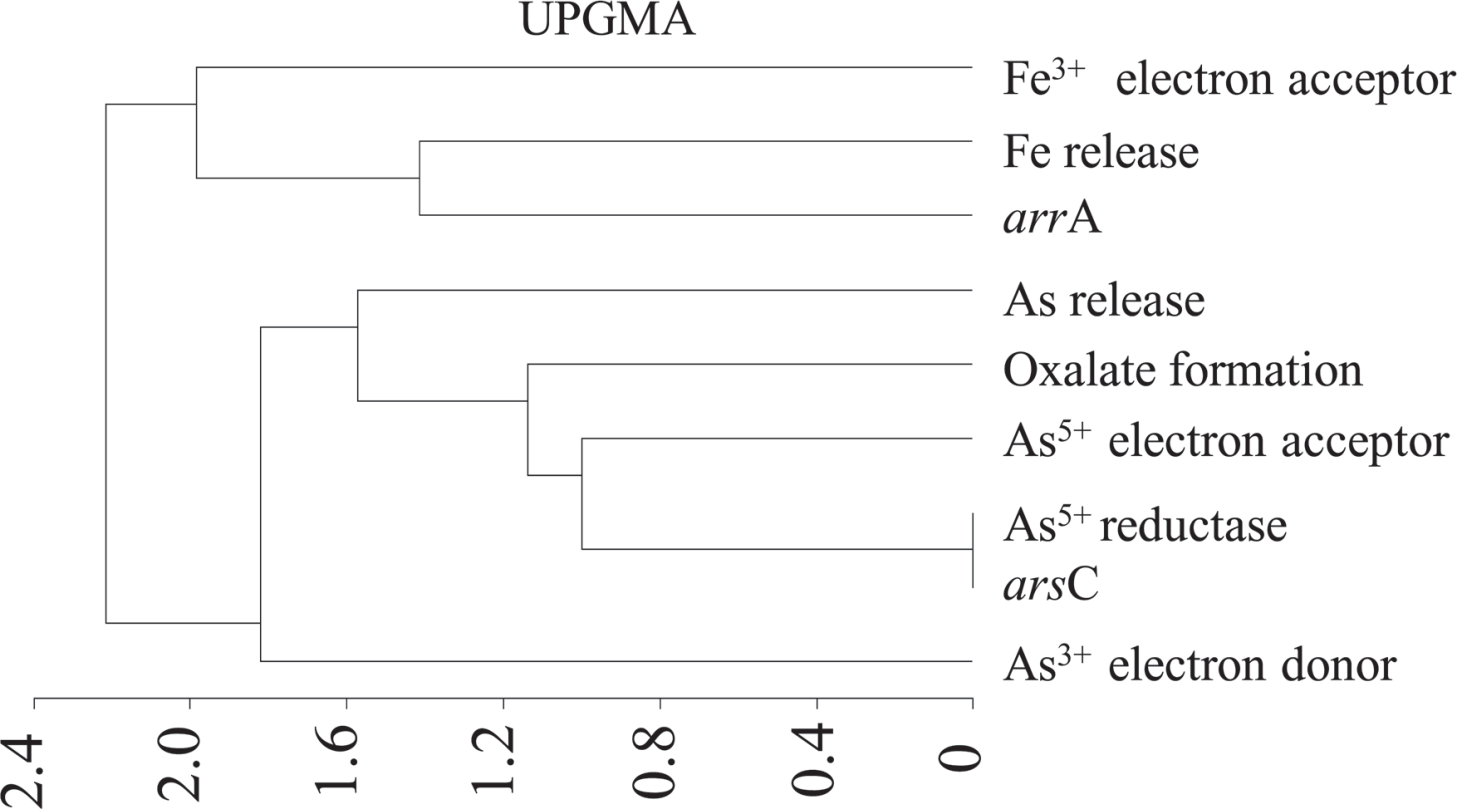
Correlation between the metabolic-genetic characteristics of bacterial strains used for microcosm study and release of As and Fe within different microcosms.

In order to get a better understanding on bacterial role in As mobilization, particularly the type of transformation taking place within the microcosms a UPGMA based analysis was performed, considering a set of metabolic and genetic characters and concentrations of As and Fe released (Fig. 11). A good agreement between As mobilization and bacterial ability to use As^5+^ as terminal electron acceptor, As^5+^ reductase activity, presence of *ars*C gene and production of oxalic acid was evident. Release of Fe, was distantly related to As release, but remained strongly correlated with bacterial ability to use Fe^3+^ as TEA and presence of *arr*A gene.

### Statistical analysis

**Fig 12:**
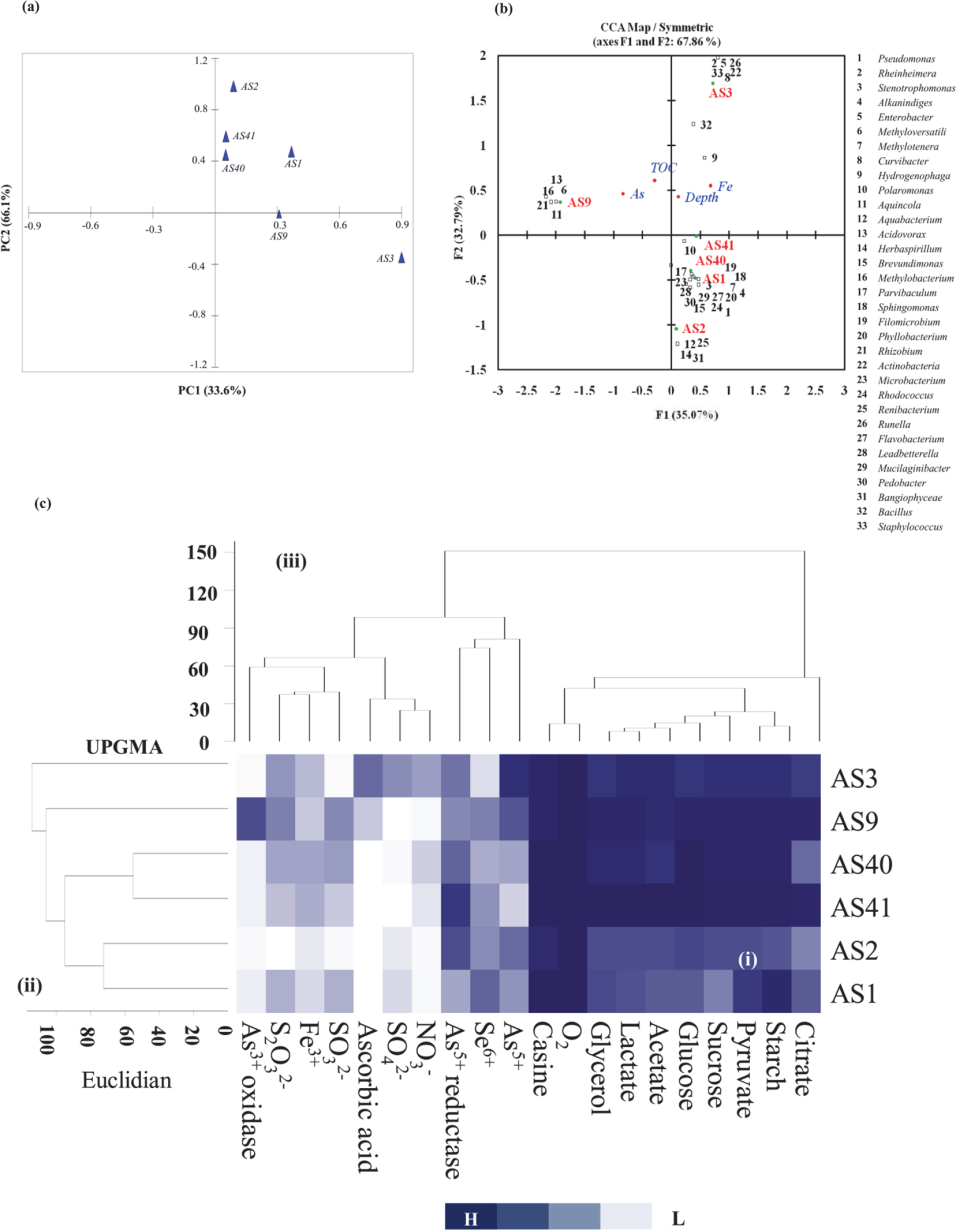
PCA, CCA and UPGMA based ordination plots on samples’ microbiological and geochemical properties. (a) Principal Component Analysis (PCA) of the samples based on geochemical parameters; (b) Canonical correspondence analysis (CCA) of samples geochemical properties and bacterial community compositions (c) ordination plots representing (i) comparative analysis of metabolic properties of the isolates obtained from six As contaminated samples, (ii) cumulative metabolic profiles represented by UPGMA for the isolates obtained from individual samples and (iii) UPGMA cluster analysis among the test metabolic properties.

Interrelation among the samples with respect to their geochemical properties, community composition and metabolic characteristics of inhabitant bacteria was studied using a number of statistical analyses. PCA performed on selected geochemical parameters indicated a resemblance between AS40, AS41, AS2 and AS1, while AS9 was distantly related to these four samples (Fig. 12A). Based on the geochemical parameters used for this analysis, AS3 remained distinctly separated from rest of the samples. In order to understand the relationship between geochemical properties and bacterial community composition (as detected by both culture-dependent and -independent approaches) Canonical Correspondence Analysis (CCA) was performed (Fig. 12B). Axes 1 and 2 of the resulting biplot accounted for 35.07% and 32.39%, respectively of total variability. The CCA data showed that a few specific groups like *Methyloversatilis*, *Methylobacterium*, *Rhizobium* and *Acidovorax* correlated to the As content. Fig. 12C represented cumulative metabolic characteristics of the strains isolated obtained from each of the samples and their interrelations. The heat map developed based on relative abundance of individual properties tested within the bacterial strains obtained from each of the samples was presented in Fig. 12C(i). The data indicated that a number of characteristics *e*.*g*., As^5+^ reductase activity, utilization of diverse carbohydrates and use of As^5+^ as TEA were more or less consistently present across the bacteria from different samples. In contrast, As^3+^ oxidase activity, ability to use Fe^3+^, NO_3_^−^ and SO_4_^2−^ as TEAs remained more sample specific. UPGMA dendogram represented that based on overall metabolic properties of indigenous bacteria the sampling sites can be divided into a few distinct clades (Fig. 12C(ii)). It could be noted that AS40 and AS41 as well as AS2 and AS1 were strongly related to each other forming two distinct clades. As also observed during geochemical parameters based analysis (Fig. 12A), samples AS9 and AS3 were distantly related to rest of the samples even with respect to metabolic properties of inhabitant bacteria. A second UPGMA was performed to analyse the interrelation between different metabolic characteristics tested within the bacterial isolates. It was evident that utilization of Fe^3+^, S_2_O_3_^2−^and SO_3_^2−^ as terminal electron acceptor was closely related, whereas ability to use NO_3_^−^ and SO_4_^2−^ was coupled together. Use of different sugars as carbon source showed higher level of similarity, and ability to use of As^5+^ or Se^6+^ as TEA was related to As^5+^ reductase activity [Fig. 12C(iii)]. Overall analyses suggest that there was a good agreement among the local geochemical parameters and composition *vis a* vis metabolic activities of indigenous bacterial groups, thereby highlighting the possible role of local geochemical conditions in shaping the bacterial communities and their metabolic characteristics.

## Discussion

Mobilization of sediment bound As into the groundwater of BDP is considered to be an outcome of complex interplay between the hydro-geo-chemical factors and microbial activities [14,54–57]. Although culture-independent and -based studies have already revealed composition of bacterial communities at a few locations of Bangladesh and West Bengal, the present study provided a better understanding of community composition and their metabolic repertoire relevant for As release in groundwater.

Physicochemical analyses of water samples were performed for better understanding of the link between bacterial community composition and function. Since the present study did not include any sediment sampling, information on lithology was gleaned from the literature [7,23,58,59]. It was found that tubewells AS1, AS3 and AS9 have accessed groundwater from Holocene and AS2, AS40 and AS41 from Pleistocene aquifer. Our analyses showed that the groundwater samples represented characteristic nature of aquifers of BDP region particularly with respect to their varying As content, reducing condition, abundant HCO_3_^−^, low DO and OC as well as lack of sufficient soluble nutrients [20–25,58,60,61]. Arsenic rich groundwater in India and Bangladesh is typically of Ca-HCO_3_ type, with high variation in As concentration on both local and regional scales [58,59]. Disparity in As level across this region is often linked with its release from ‘a disperse As source’ (*i*.*e*., not anthropogenic point sources) [58]. Overall, the chemical state of the groundwater represents an oligotrophic condition for the indigenous microorganisms. Sediment and associated groundwater of Holocene aquifer are known to be maintaining reducing condition with gray sand capped by soft organic matter-rich clay unit making them semi-confined to leaky confined [7,21]. Although dissolution of As from Fe/Mn oxides/hydroxides based host minerals of this gray sand is considered to be a major source of soluble As, its concentration in groundwater often showed no strong correlation with that of Fe [7,59]. The same (absence of strong correlation between As and Fe) has also been observed in our study and supports the hypothesis that mechanisms for As and Fe release are not necessarily coupled [57,58].

Using a combination of culture-dependent and -independent studies we succeeded to identify presence of 39 genera within the samples and out of these 21 had never been reported from As rich sites of Bangladesh or West Bengal. Our observation highlights the complementary nature of both these approaches and at the same time substantiated the fact that the range of microbial diversity captured by culture-independent approach can be extended by parallel application of culture based method as well [61–63]. Previous studies have only highlighted presence of complex, often inconsistent community composition with dominance of one or few aerobic, facultative anaerobic, chemolithotropic, nitrate reducing or obligate anaerobic genera [9,14,15,20,21]. The present study establishes that in spite of elevated As and poor nutrient contents, bacterial communities in As rich groundwater can be significantly diverse. A level of consistency in community composition is also revealed by frequent detection of several genera within the samples from different locations and As content. With respect to diversity of genera detected, *Mucilaginibacter* was neither previously reported from As rich aquifers, nor have any known interaction with As and therefore noticed for the first time in this study. Some other groups *e*.*g*., *Alkanindiges*, *Enterobacter*, *Methylotenera*, *Methylobacterium*, *Polaromonas*, *Rhodococcus*, *Rheinheimera* and *Stenotrophomonas* though have been reported earlier from As rich environments [21] but their metabolic properties relevant for survival and function in such contaminated niches remained unexplored. Abundance and As homeostasis mechanisms of several genera (*e*.*g*., *Rhizobium*, *Brevundimonas*, *Pseudomonas*, *Enterobacter*, *Acidovorax*, *etc*.) as detected in our samples, however, are well known [21,26].

Our study on metabolic characterization of the isolated bacterial strains highlighted that groundwater bacterial communities are not only constituted by assemblages of taxonomically diverse populations, but they also possess a very robust metabolic potential as well. Abundance of As-resistance and -transformation properties, particularly the presence of both As^5+^ reducing and As^3+^ oxidizing activities and elevated As^3+^/As^5+^ resistance under aerobic and anaerobic conditions could possibly indicate the essentiality of such traits within the autochthonous populations for their survival and activity in As rich environment. Co-occurrence of As oxidizing and reducing activities within the bacterial populations present in the same niche is considered to be an evolutionary outcome facilitating survival of inhabitant bacteria in shallow aquifer systems [29,31]. As evident during this study, As resistance and transformation properties were in good agreement with the presence of relevant As homeostasis genes (*e*.*g*., *ars*C and *acr3*(2)). With respect to As resistance, it was also noted that ability to withstand higher As^3+^ and/or As^5+^ concentrations was strongly supported by As^3+^ oxidase gene (*aio*B) or cytosolic As^5+^ reductase (*ars*C) gene. Simultaneous presence of As^3+^ efflux (*arsB* and *acr*3(2)) and *ars*C genes further highlighted the strong genetic makeup of the bacteria towards complete detoxification mechanism. It is important to note that along with As homeostasis systems spread through the As concentration gradient of different samples, the bacterial isolates showed high level of metabolic diversity. Their abilities to utilize multiple carbohydrates, complex hydrocarbons and even HCO_3_ during heterotrophic or autotrophic metabolism, use of As^3+^ as electron donor and inorganic electron acceptors during anaerobic growth clearly demonstrate the metabolic flexibility to sustain life under different nutritional conditions. Arsenic rich alluvial aquifer of western part of Bengal delta (West Bengal) is oligotrophic in nature with low dissolve carbon and varied oxygen content. Together with higher amount of bicarbonate, presence of low concentration of petroleum derived hydrocarbons naturally seeping into shallow aquifer from deeper, more thermally mature sediments have been previously reported [15, 24]. Petroleum hydrocarbons can be metabolized through a variety of processes and in particular, their biodegradation under reducing condition is very well known [24]. Study on anaerobic metabolism of *n-*alkane utilizing As^5+^ as TEA by selected bacterial strains obtained from As rich groundwater provided confirmatory evidence on such abilities (data not shown). Although in general As contaminated groundwater of West Bengal maintains a nearly consistent geo-chemical character, dynamic and local fluctuations in depth distribution and associated reconfiguration of redox conditions could potentially affect the availability of electron donors and acceptors and their use by inhabitant bacteria. Considering the overall hydro-geo-chemical dynamics of groundwater, evolution of a broader metabolic repertoire within the autothonous bacteria capable of utilizing diverse respiratory substrates and carry out redox transformation of As species seems highly justified.

Based on metabolic properties either observed or derived from phylogenetic lineages bacterial populations could be categorized in a number of functional groups represented by specific populations: (i) heterotrophic bacteria capable of aerobic as well as anaerobic metabolism utilizing multiple electron donors and acceptors (*e*.*g*., *Pseudomonas*, *Bacillus*, *Staphylococcus*, *Acinetobacter* and *Stenotrophomonas*); (ii) hydrocarbon metabolizing organisms with or without marine lineage (*e*.*g*., *Polaromonas* and *Rhodococcus*); (iii) C1 compound utilizing facultative anaerobic bacteria (*e*.*g*., *Methylotenera* and *Methyloversatilis*); (iv) organisms with chemolithotropic or chemolithoautotrophic activity (*e*.*g*., *Brevundimonas*, *Hydrogenophaga* and *Rhizobium*) and (v) bacteria capable of mineral weathering (*e*.*g*., *Arthrobacter* and *Pseudomonas*). Predominance of *Pseudomonas*, *Acinetobacter*, *Bacillus* and *Staphylococcus* had previously been reported in As contaminated alluvial Bengal delta [9,14,15,20,21,27]. Members of these genera are well known for their capacity to withstand high As concentration [9,20,21]. Metabolic robustness of these genera as evident from their capacity to grow aerobically and anaerobically utilizing diverse electron/carbon donors and acceptors corroborates very well with earlier reports indicating their ‘ability to live at the interface of aerobic and anaerobic environment’ [20,21]. Such abilities allow these organisms not only to play important role in As biogeochemistry but also create anoxic/reducing conditions within the As rich environment [1,21,15,63,64]. Hydrocarbon utilizing marine *Polaromonas* had not been previously reported from BDP aquifers. Along with *Polaromonas*, abundance of another hydrocarboncladstic genus *Rhodococcus* (as culturable member) was an important observation. Both these genera are well known for their abilities to utilize petroleum hydrocarbons, survive with elevated As and even nutrient limiting condition [65]. Presence and abundance of these two genera are consistent with the nature of BDP aquifer. Though the alluvial As contaminated groundwater of BDP often showed low bulk OC content, presence of low concentration of petroleum derived hydrocarbons have been reported earlier [25]. Ability of many of our isolated strains to utilize diverse hydrocarbon compounds *e*.*g*., cyclohexane, nonadecane, docosane, *etc*. as sole carbon source, supports such view that inhabitant bacteria in As contaminated aquifers indeed are capable of metabolizing the hydrocarbon compounds. Though the presence (concentration) and nature of petroleum derived organic matter do not behave ‘conservatively’ within the As rich aquifers of South East Asia, nevertheless it is known to be utilized by indigenous microorganisms to fuel microbe mediated As release [66]. Biodegradation of petroleum hydrocarbon in reducing aquifer is well known indicating further that these hydrocarbons could potentially act as electron donor for Fe/As reduction and subsequent As release in shallow aquifer. The third abundant group *i*.*e*., C1-, as well as multicarbon compound utilizing facultative anaerobic bacteria affiliated to genera *Methyloversatilis*, *Methylotenera*, *Flavobacterium* and *Methylobacterium* as detected during this study have been reported by previous investigators [67]. Presence of methane in groundwater of BDP aquifer is well known [68] and therefore abundance of such methane utilizing bacteria in groundwater justifies their occurrence. Bacterial genera including *Brevundimonas*, *Rhizobium*, *Hydrogenophaga* and *Herbaspirillum* are well known for their chemolithoautotrophic mode of metabolism [25,27]. Although often isolated as heterotrophs, these organisms can use energy and reducing power from oxidation of various inorganic elements including As^3+^/Fe^2+^/Mn^2+^ during CO_2_ fixation or other anabolic reactions and grow under aerobic or anaerobic oligotrophic environments [27]. Several bacterial strains affiliated to these genera have been reported recently from As rich sites of BDP and other places [25,26,69]. Members of genera *Polaromonas*, *Arthrobacter*, *Aquabacterium*, *Pseudomonas*, *Rhizobium*, *Bacillus*, *Staphylococcus* and *Acinetobacter* as detected in our samples have previously been reported for their weathering activity. Production of siderophore, oxalic acid and/or gluconic acid by most of these bacteria as major agents for nutrient acquisition have been observed [70,71].

In low organic carbon containing aquifers of BDP microbially mediated redox transformation that lead to release of sediment bound As is considered to be regulated by availability of organic carbon and catabolic potential of bacterial species present. Decoupling of As and Fe release and involvement of cytosolic As^5+^ reductase activity over the dissimilatrory metal reductase have been reported [22,15,9,31]. The present microcosm study showed that As^5+^ reducing bacterial strains with or without dissimilatory As^5+^/Fe^3+^ reduction ability and *arr*A gene could mobilize As within the aqueous phase. Together with As, co-release of several other elements and concomitant mineralogical changes in the incubated sand indicated possible dissolution of constituting minerals [*viz*, Fe/Mn (Al) oxides/hydroxides] leading to mobilization of these elements. Though the concentrations of aqueous As did not maintain any strong correlation with Fe and other elements released, the level of oxalate and As remained in good agreement. It is also important to note that none of the strains used in these microcosms was affiliated to known dissimilatory As/Fe reducing genera, nevertheless, a few strains (*Rhodococcus* CAS922i and *Brevundimonas* CAS4005i) possessing *arr*A gene and ability to use As^5+^ as TEA could mobilize higher concentration of As. The level of released As and its species (as As^3+^) in microcosms with these bacteria corroborated very well with earlier studies, wherein As bearing sand containing indigenous microbial populations or sterile sand exposed to Fe reducing *Shewanella* sp. was used in microcosm [15,57]. Presence of As^3+^ in aqueous phase from the early stage of incubation (24h onward) indicated that subsequent to its release from the host mineral, As^5+^ gets reduced by bacterial reductases, which could either be for detoxification or energy generation. While the dissmilatory As^5+^ reduction process might play an important role in case of some bacteria, the observed good agreement among presence of *ars*C gene, As^5+^ reductase activity, oxalate production and As mobilization clearly indicates a more complex interplay of bacterial catabolic abilities. Within the microcosms changes detected in sediment mineralogy/composition suggest that bacterial strains are able to cause dissolution of As bearing sand and thereby facilitating mobilization of such elements. This dissolution could be caused by different metabolites *e*.*g*., organic acids, siderophore, cyanides, *etc*. often released by this bacteria [13,50]. Although siderophore production was not so frequently detected within the test bacteria, presence of oxalic acid and its correlation with As release certainly highlights the role of such event. Considering the overall characteristics of the bacterial strains we hypothesize that bacterial strains metabolically equipped for weathering of minerals and As reduction cause dissolution of the host minerals in search of their nutrient(s). This dissolution results sediment associated As^5+^ to be dislodged with co-release of other elements. The released As^5+^ get reduced to As^3+^ as the bacteria use it either as TEA or transform it for detoxification.

### Conclusions

Overall study indicates that bacterial communities in As rich groundwater of West Bengal are constituted by diverse taxonomic groups, metabolically as well as genetically well equipped to survive and function within the aquifer environment. Arsenic mobilization in the aquifer may be an outcome of collective metabolic reactions of these indigenous populations. Unlike preponderance of any particular event, release of sediment bound As could possibly occurred due to a cascade of catabolic processes namely dissolution of host minerals, followed by bacterial As^5+^ reducing activities or direct action of bacterial reductive machinery on host minerals resulting elevated As^3+^ in aqueous phase. These processes are not mutually exclusive but may incorporate each other. Furthermore, our orange sand based microcosm study indicates that significant mobilization of As can occur, provided suitable bacterial strains interact with them. So conclusively, though the present perception reveals relatively safe nature of orange sand (Pleistocene aquifer) as a source of As free drinking water our study indicates its potential vulnerability.

## Supporting Information

S1 Fig Rarefaction curves for bacterial operational taxonomic units (OTUs) retrieved from six As contaminated groundwater samples. (TIF)

S2 Fig DGGE profile of PCR amplified 16S rRNA gene fragments. Bands excised and analysed further are shown by underlined numerals.
(TIF)

S3 Fig Concentration of oxalic acid present in aqueous phase of microcosms (after 300 days). Error bars indicate standard deviations (n = 3). The bacterial strains represented in X axis are as follows: CAS922i (*Rhodococcus* sp.), CAS4005i (*Brevundimonas* sp.), BAS108i (*Staphylococcus* sp.), BAS224i (*Phyllobacterium* sp.), CAS4101i (*Arthrobacter* sp.), BAS323i (*Pseudomonas* sp.), BAS123i (*Acinetobacter* sp.) and CAS907i (*Pseudomonas* sp.).
(TIF)

S1 Table Details of the genes targeted for PCR and their respective primers. (PDF)

S2 Table Details of the metabolic characters used in Eucladian biplot analysis of the selected bacterial isolates. (PDF)

S3 Table Trace elements detected within the As contaminated groundwater samples (ICP-MS analysis). (PDF)

S4 Table Correlation between As and Fe, Mn, SO4, NO3, HCO3, TOC, and CFU in the groundwater, collected from Barasat and Chakdaha, West Bengal. (PDF)

S5 Table Designations of the isolates corresponding to the numbers allotted in the PCA plot. (PDF)

S6 Table Microbial counts during 300 days incubation in microcosm experiment. (PDF)

S7 Table Elemental composition of the orange sand before and after 300 days incubation with selected bacterial strains under anaerobic condition. (PDF)

S8 Table Correlation between As and Mn, Fe, Na, Ca, K and Al in the aqueous phase of microcosm after 300 days incubation. (PDF)
