# Supplementary figures and images for: Diversity, Metabolic Properties and Arsenic Mobilization Potential of Indigenous Bacteria in Arsenic Contaminated Groundwater of West Bengal, India
Source: PLoS One. 2015 Mar 23;10(3):e0118735. doi: 10.1371/journal.pone.0118735 (PMC4370401; doi:10.1371/journal.pone.0118735)

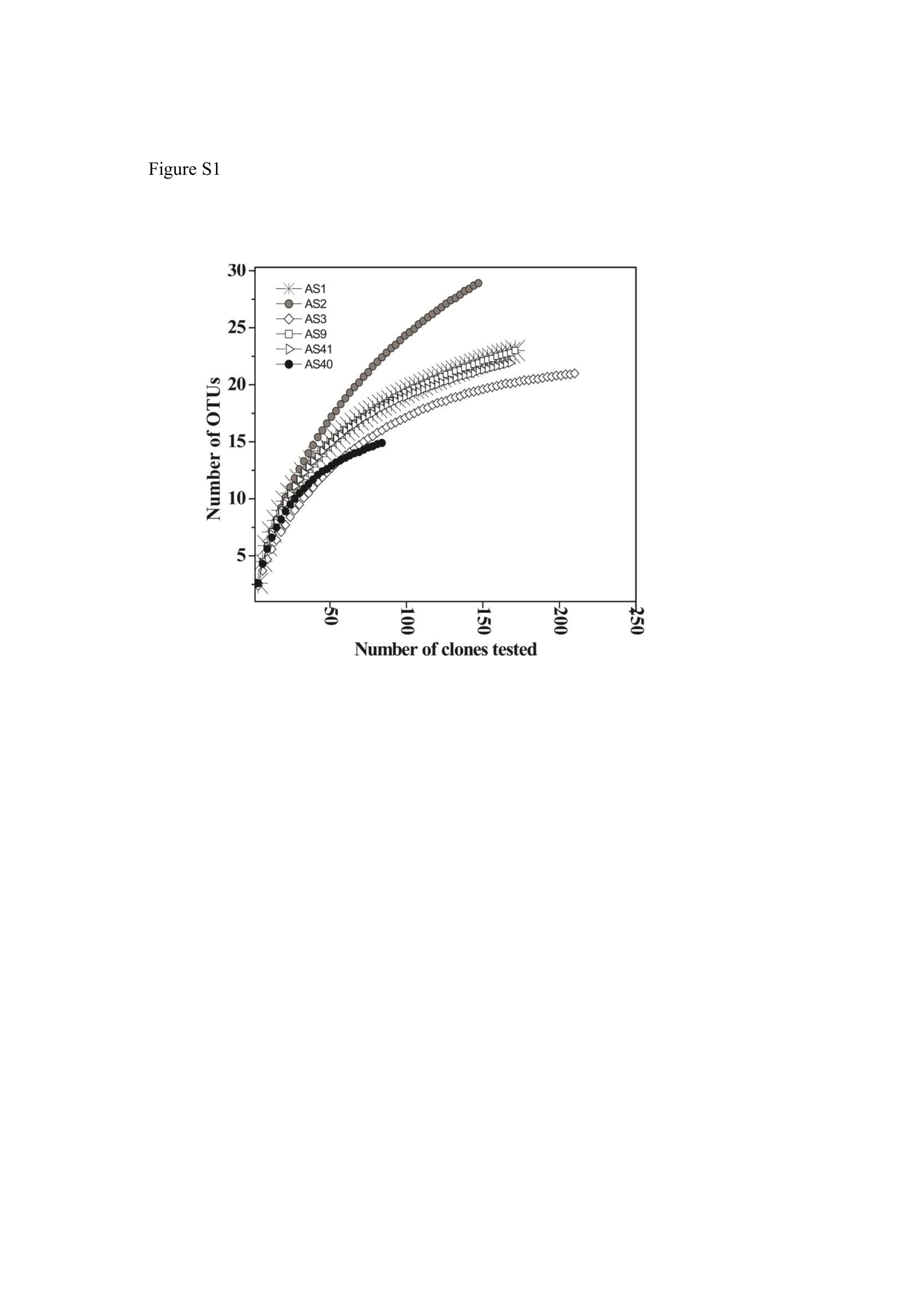

Supplement: S1 Fig — (TIF) [file pone.0118735.s001.tif]

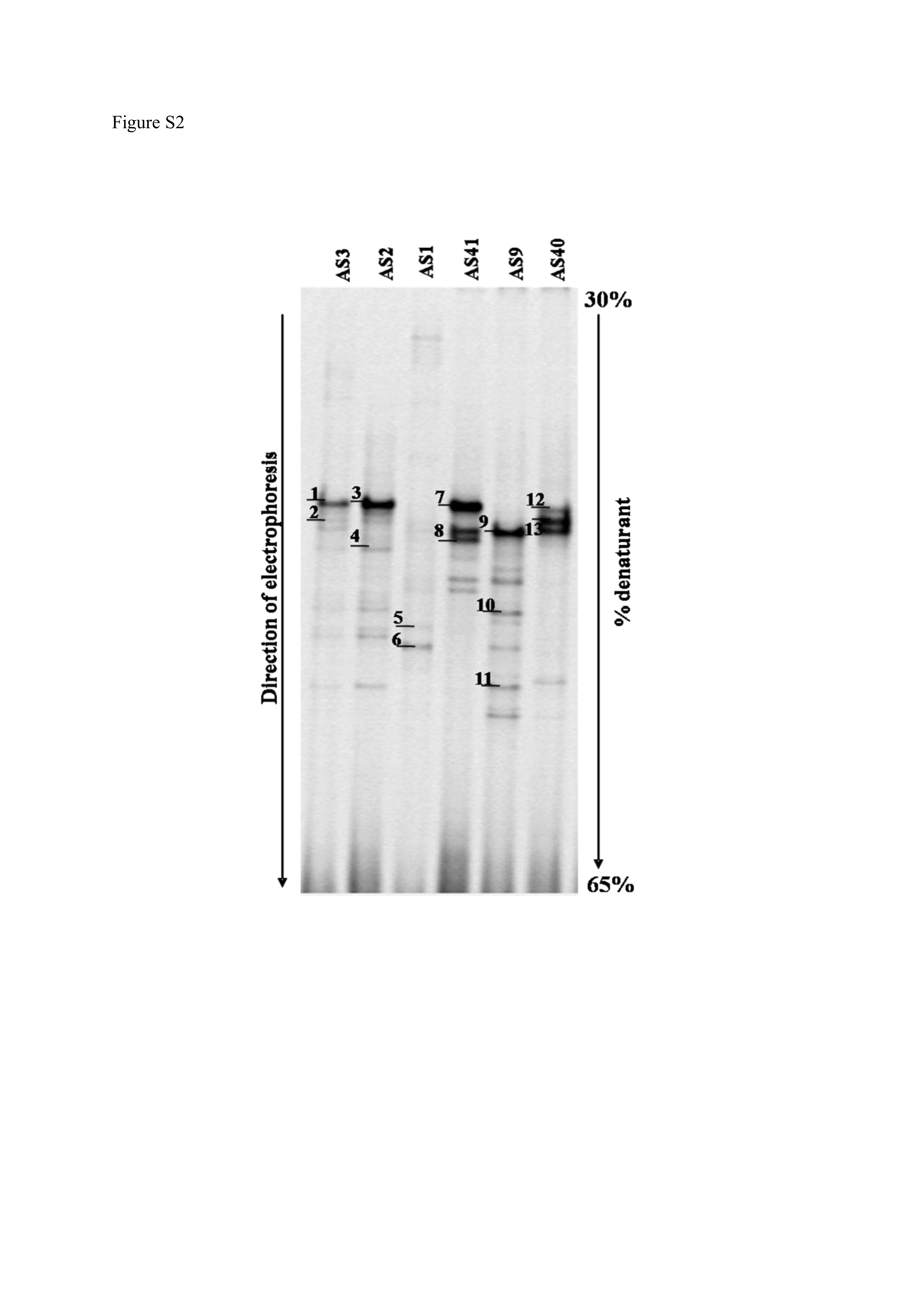

Supplement: S2 Fig — Bands excised and analysed further are shown by underlined numerals. (TIF) [file pone.0118735.s002.tif]

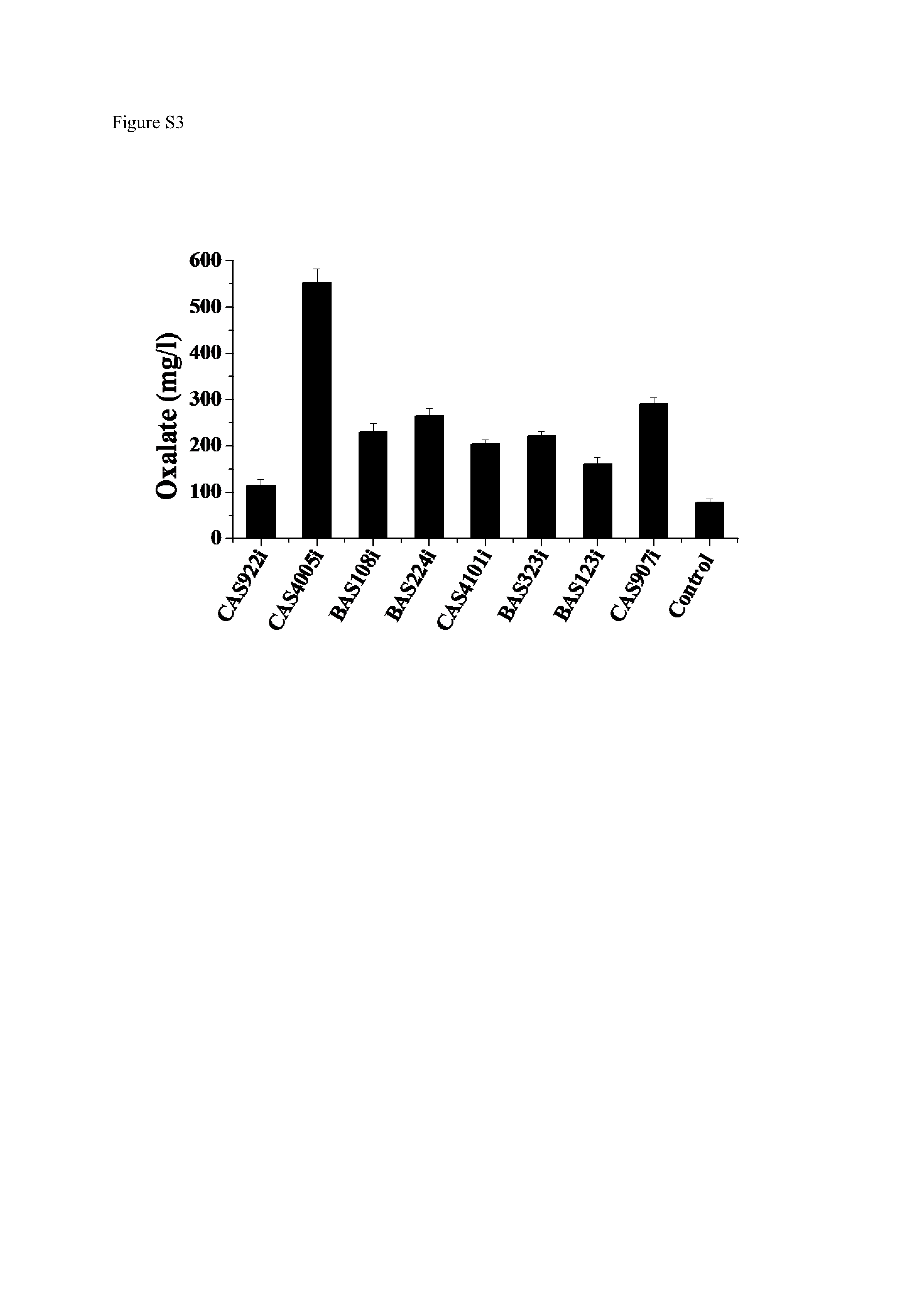

Supplement: S3 Fig — Error bars indicate standard deviations (n = 3). The bacterial strains represented in X axis are as follows: CAS922i (Rhodococcus sp.), CAS4005i (Brevundimonas sp.), BAS108i (Staphylococcus sp.), BAS224i (Phyllobacterium sp.), CAS4101i (Arthrobacter sp.), BAS323i (Pseudomonas sp.), BAS123i (Acinetobacter sp.) and CAS907i (Pseudomonas sp.). (TIF) [file pone.0118735.s003.tif]
