# Supplementary material for: Diversity, Metabolic Properties and Arsenic Mobilization Potential of Indigenous Bacteria in Arsenic Contaminated Groundwater of West Bengal, India
Source: PLoS One. 2015 Mar 23;10(3):e0118735. doi: 10.1371/journal.pone.0118735 (PMC4370401; doi:10.1371/journal.pone.0118735)
Supplement: S1 Table — (PDF) [file pone.0118735.s004.pdf]

Table S1 Details of the genes targeted for PCR and their respective primers

Gene	Description	Primer sequence	Product size (bp)	PCR condition	Reference
16S rRNA	Ribosomal RNA gene	27 F: 5-AGAGTTTGATCMTGGCTCAG-3<br>1492R: 5-GGTTACCTTGTTACGACTT-3	1500	94 ºC (5min)<br>94 ºC (45s)<br>58 ºC (30s) } 30cycle<br>72 ºC (90s)<br>72 ºC (10min)<br>4 ºC (infinite)	Islam and Sar (2011)
16s rRNA	V3 region of 16S rRNA genes	5'-CGCCCGCCGCGCCCCGCGCCCGGCCCGCCG<br>CCCCCGCCCCCCTACGGGAGGCAGCAG-3'<br>5'-ATTACCGCGGCTGCTGG-3'	˜177	95 °C (5min)<br>94 °C (30s)<br>61 °C (-0.5°C) (30s) } 10 cycle<br>72 °C (5s)<br><br>94 °C (30s)<br>56 °C (30s) } 25 cycle<br>72 °C (45s)<br>72 °C (7min)<br>4 ºC (infinite)	Muyzer et al. (1993).
*ars*C	Cytosolic arsenate reductase	amlt-42 F: 5-TCGCGTAATACGCTGGAGAT-3<br>amlt-376 R: 5-ACTTTCTCGCCGTCTTCCTT-3	390	94 ºC (5 min)<br>94 ºC (30s)<br>54 ºC (30s) } 30cycle<br>72 ºC (30s)<br>72 ºC (10min)<br>4 ºC (infinite)	Sun et al. (2004)
*ars*C	Cytosolic arsenate reductase	smrc-42 F: 5-TCACGCAATACCCTTGAAATGATC-3<br>smrc-376 R: 5-ACCTTTTCACCGTCCTCTTTCGT-3	390	94 ºC (5 min)<br>94 ºC (30s)<br>59 ºC (30s) } 30 cycle<br>72 ºC (30s)<br>72 ºC (10min)<br>4 ºC (infinite)	Sun et al. (2004)
*aio*B	Periplasmic arsenite oxidase	AoxB-F1: 5-ACVTTCAASTGYCCHKGYCAYTTC-3<br>AoxB-R1: 5-TGRTTNAGRAARTARTTNGTYTG-3	650	94 ºC (5min)<br>94 ºC (45s)<br>57-52 ºC (-0.5 ºC)(45s) } 10 cycle<br>72 ºC (30s)	Inskeep et al. (2008)

		AoxB-F2: 5-TGYCAYTTYTGYATHGTNGGNTG-3<br>AoxB-R2:5-TANGCNGGNCGRTTRTGDAT-3		94 ºC (45s)<br>57 ºC (45s) } 25 cycle<br>72 ºC (30s)<br>72 ºC (10 min)<br>4 ºC (infinite)	
*acr*3(2)	Arsenite efflux pump	dacr5F 5: TGATCTGGGTCATGATCTTCCCVATGMTGVT-3<br>dacr4R 5:-CGGCCACGGCCAGYTCRAARAARTT-3	650	94 ºC (5min)<br>94 ºC (45s)<br>57-52 ºC(-0.5 ºC) (45s) } 10 cycle<br>72 ºC (30s)<br><br>94 ºC (45s)<br>57 ºC (45s) } 25 cycle<br>72 ºC (30s)<br>72 ºC (10 min)<br>4 ºC (infinite)	Achour et al. (2007)
*ars*B	Arsenite efflux pump	dacr1F 5-GCCATCGGCCTGATCGTNATGATGTAYCC-3<br>darsB1R: 5-CAGGCCGTACACCACCAGRTACATNCC-3	650	94ºC (5min)<br>94ºC (45s)<br>57-52 ºC (-0.5 ºC) } 10 cycle<br>72 ºC (30s)<br><br>94 ºC (45s)<br>57 ºC (45s) } 25 cycle<br>72 ºC (30s)<br>72 ºC (10 min)<br>4 ºC (infinite)	Achour et al. (2007)
*arr*A	Respiratory arsenate reductase	arrA-as1-F 5-CGAAGTTCGTCCCGATHACNTGG-3<br>arrA-as1-R 5'-GGGGTGCGGTCYTTNARY TC-3<br>arrA-as2-F 5-GTCCCNATBASNTGGGANRARGCN MT-3<br>arrA-as2-R 5-ATANGCCCARTGNCCYTGNG-3	630<br><br>2000	**PCR I**<br>94 ºC (5 min)<br>94 ºC (30s)<br>50 ºC (30s) } 35 cycle<br>72 ºC (1 min)<br>72 ºC (10min)<br>4 ºC (infinite)<br><br>**PCR II**<br>94 ºC (5 min)<br>94 ºC (30s)<br>55 ºC (30s) } 35 cycle<br>72 ºC (2 min)	Song et al. (2009)

			625	72 ºC (10min)<br>4 ºC (infinite)<br><br>**PCR III**<br>94 ºC (2 min)<br>94 ºC (30s) } 30 cycle<br>55 ºC (30s)<br>72 ºC (1 min)<br>72 ºC (10min)<br>4 ºC (infinite)	

B G, T, or C; *M* A or C; *N* A, C, G, or *T*; R A or G; *S* G or C; *V* A, C, or G; *Y* C or T
