# Supplementary material for: Diversity, Metabolic Properties and Arsenic Mobilization Potential of Indigenous Bacteria in Arsenic Contaminated Groundwater of West Bengal, India
Source: PLoS One. 2015 Mar 23;10(3):e0118735. doi: 10.1371/journal.pone.0118735 (PMC4370401; doi:10.1371/journal.pone.0118735)
Supplement: S2 Table — (PDF) [file pone.0118735.s005.pdf]

Table S2: Details of the metabolic characters used in Eucladian biplot analysis of the selected bacterial isolates (Figure 9b).

	As^{3+} resistance	As^{5+}resistance	Nonadecane	Docosane	Dodecane	Pentadecane	Cyclohexane	Phenanthrene	Naphthalene	Pyrene	Fluorene	Anthracene	NaHCO3	As31 electron donor	Motility	Siderophore	*ars*B	*ars*C	*acr*3(2)	*aio*B	*arr*A	As^{3+} oxidase	As^{5+} reductase
Acinetobacter BAS123i	1	1	1	1	1	1	1	1	1	1	1	1	1	1	0	0	1	1	0	1	0	1	1
Arthrobacter CAS4117i	1	1	1	1	1	1	1	1	1	1	1	1	1	1	1	0	0	0	0	0	0	0	1
Arthrobacter CAS4101i	1	1	1	0	1	1	1	1	1	1	1	1	1	1	1	1	1	1	1	0	0	0	1
Bacillus BAS204i	0	0	0	0	0	0	0	0	0	0	0	0	1	0	1	0	0	0	0	0	0	1	1
Brevundimonas BAS230i	0	0	1	1	1	1	1	1	1	1	1	1	1	1	1	0	1	1	0	0	0	0	0
Brevundimonas CAS4119i	0	0	1	1	1	1	1	1	1	1	1	1	1	0	1	0	0	1	0	0	0	0	1
Brevundimonas CAS4123i	0	1	1	1	1	1	1	1	1	1	1	1	1	0	1	0	1	1	0	0	0	0	1
Brevundimonas CAS4008i	1	0	1	1	1	1	1	1	1	1	1	1	1	0	1	0	1	0	0	0	0	1	0
Brevundimonas BAS223i	1	0	1	1	1	1	1	1	1	1	1	1	1	0	1	0	1	0	0	0	0	1	1
Brevundimonas CAS4005i	1	1	1	0	1	0	1	1	1	1	1	1	1	0	1	0	1	1	1	0	1	0	1
Herbaspirillum CAS4110i	0	0	1	1	1	1	1	1	1	1	1	1	1	0	1	0	0	0	0	0	0	0	0
Hydrogenophaga CAS4014i	1	1	1	1	1	1	1	1	1	1	1	1	0	1	1	0	0	0	0	0	0	0	0
Microbacterium CAS905i	1	1	1	1	0	1	1	1	1	1	1	1	0	1	1	0	1	0	0	1	0	1	1
Phyllobacterium BAS211i	1	1	0	0	1	1	0	0	1	1	0	1	0	0	1	0	0	0	0	0	0	0	1
Phyllobacterium BAS224i	1	1	1	0	1	1	1	1	1	1	1	1	1	0	0	0	1	1	0	0	0	0	1
Pseudomonas CAS934i	1	1	1	1	1	1	1	1	1	1	1	1	1	1	1	0	1	0	0	0	0	0	1
Pseudomonas CAS4116i	1	0	1	1	1	1	1	1	1	1	1	1	1	0	1	0	0	1	1	0	0	0	1
Pseudomonas CAS4106i	0	0	1	1	1	1	1	1	1	1	1	1	1	0	1	0	0	1	1	0	1	0	1
Pseudomonas CAS4105i	0	0	1	1	1	1	1	1	1	1	1	1	1	1	0	0	1	0	0	0	0	0	0
Pseudomonas BAS309i	0	0	1	0	1	0	1	1	1	1	1	1	1	0	1	0	0	1	1	0	0	0	0
Pseudomonas CAS4016i	1	1	1	1	1	1	1	1	1	1	1	1	0	0	1	0	1	0	0	0	0	0	1
Pseudomonas CAS908i	1	1	1	0	1	1	0	1	1	0	0	1	1	0	1	0	1	1	0	0	1	0	1
Pseudomonas CAS4001i	1	1	0	0	1	0	1	1	1	1	1	0	1	0	1	1	1	0	0	0	1	0	1
Pseudomonas BAS323i	1	1	1	1	1	1	1	1	1	1	1	1	1	1	0	0	1	1	0	0	0	0	1
Pseudomonas CAS907i	1	1	1	0	0	1	1	1	1	0	1	1	1	1	0	0	1	1	1	0	1	0	1
Rheinheimera BAS124i	0	1	1	0	1	1	1	1	1	1	1	1	1	0	1	0	0	0	0	0	0	0	1
Rheinheimera BAS122i	1	0	1	0	0	1	1	1	1	0	1	1	1	0	1	0	0	1	0	0	0	0	1
Rheinheimera BAS127i	0	1	1	1	1	1	1	1	1	1	1	1	0	0	1	0	0	0	0	0	0	0	0
Rhizobium CAS325i	1	1	1	1	1	1	1	1	1	1	1	1	1	0	1	0	0	1	1	0	0	0	1
Rhizobium BAS306i	1	1	1	0	1	1	1	1	1	1	1	1	0	0	1	0	1	1	1	0	0	0	1
Rhizobium CAS4026i	1	1	1	0	1	1	1	1	1	1	1	1	1	1	1	0	0	0	1	0	0	0	0
Rhizobium CAS4022i	1	1	1	1	1	1	1	1	1	1	1	1	1	0	0	0	1	0	0	0	0	0	1
Rhizobium BAS310i	0	1	1	1	1	1	1	1	1	1	1	1	1	1	0	0	0	0	1	0	0	0	1
Rhizobium BAS316i	0	0	1	0	1	1	1	1	0	1	0	1	1	0	0	0	0	1	1	0	0	0	0
Rhizobium BAS305i	0	0	1	0	1	1	1	1	1	1	1	1	1	0	0	0	0	1	0	0	0	0	0
Rhodococcus CAS912i	1	0	1	1	1	1	1	1	1	1	1	1	1	1	0	0	1	0	0	0	0	0	0
Rhodococcus CAS931i	1	1	1	1	1	1	1	1	1	1	1	1	1	1	0	0	1	0	0	0	0	1	0
Rhodococcus CAS930i	1	1	1	0	1	1	1	1	1	1	1	1	1	1	0	0	0	0	1	0	1	1	1
Rhodococcus CAS933i	1	1	1	0	1	1	1	1	0	1	1	1	0	1	0	0	1	0	0	1	0	1	1
Rhodococcus CAS4021i	1	1	0	0	1	1	1	0	1	0	0	0	0	0	1	1	0	0	0	0	0	1	0
Rhodococcus CAS922i	1	1	1	0	1	1	1	1	1	1	1	1	1	1	0	0	1	1	0	1	1	1	1
Staphylococcus CAS106i	1	1	1	0	1	1	1	1	1	1	1	1	1	0	0	0	1	1	1	1	0	0	1
Staphylococcus BAS108i	1	1	1	1	1	1	1	1	1	1	1	1	1	0	0	0	1	1	1	0	0	0	1
Stenotrophomonas BAS202i	0	0	0	0	0	0	0	0	0	0	0	0	1	0	1	0	0	0	0	0	0	0	0

Presence or absence of a metabolic character was marked with 1 or 0, respectively.
