# Supplementary material for: Diversity, Metabolic Properties and Arsenic Mobilization Potential of Indigenous Bacteria in Arsenic Contaminated Groundwater of West Bengal, India
Source: PLoS One. 2015 Mar 23;10(3):e0118735. doi: 10.1371/journal.pone.0118735 (PMC4370401; doi:10.1371/journal.pone.0118735)
Supplement: S3 Table — (PDF) [file pone.0118735.s006.pdf]

Table S3. Trace elements detected within the As contaminated groundwater samples (ICP-MS analysis)

Elements	**Sample ID**					
(μg/l)	***AS1***	***AS2***	***AS3***	***AS9***	***AS40***	***AS41***
Li	4.384	3.145	4.239	3.037	7.708	4.403
Al	46.985	9.341	63.766	33.224	32.903	41.945
Sc	3.898	4.273	3.908	3.500	4.204	4.113
V	0.862	1.026	1.010	0.743	1.675	0.713
Cr	3.130	3.414	3.245	2.680	1.978	2.670
Co	0.910	0.437	0.368	0.567	0.339	0.442
Ni	6.802	6.734	7.309	5.961	4.804	5.701
Zn	38.289	141.900	31.740	108.180	45.610	318.610
Rb	2.090	2.798	1.227	4.623	0.534	1.925
Ag	0.016	0.007	0.010	0.007	0.004	0.020
Cd	0.043	0.021	0.076	0.044	0.030	0.070
Ba	286.763	164.623	42.672	153.370	59.573	161.331
La	0.033	0.039	0.043	0.026	0.018	0.040
Ce	0.053	0.075	0.089	0.049	0.035	0.071
Pr	0.005	0.011	0.007	0.004	0.003	0.004
Nd	0.015	0.032	0.016	0.011	0.007	0.012
Sm	0.004	0.006	0.004	0.003	0.002	0.003
Eu	0.136	0.074	0.019	0.067	0.027	0.068
Gd	0.006	0.009	0.005	0.004	0.002	0.005
Tb	0.001	0.001	0.001	0.001	0.000	0.001
Dy	0.002	0.005	0.002	0.002	0.001	0.002
Ho	0.001	0.001	0.001	0.000	0.001	0.001
Er	0.001	0.003	0.002	0.001	0.003	0.002
Tm	0.001	0.001	0.000	0.000	0.001	0.000
Yb	0.001	0.003	0.002	0.001	0.004	0.002
Lu	0.000	0.001	0.000	0.000	0.001	0.000
Hf	0.149	0.079	0.074	0.137	0.063	0.098
Ta	0.006	0.007	0.011	0.012	0.007	0.005
W	11.064	76.934	16.507	26.293	10.648	18.317
Ir	0.894	0.537	1.351	2.429	2.398	1.778
Pb	0.956	1.928	1.432	0.863	0.714	3.294
Th	0.003	0.006	0.001	0.001	0.002	0.001
U	0.024	0.016	0.187	0.023	0.241	0.013
