# Supplementary material for: Diversity, Metabolic Properties and Arsenic Mobilization Potential of Indigenous Bacteria in Arsenic Contaminated Groundwater of West Bengal, India
Source: PLoS One. 2015 Mar 23;10(3):e0118735. doi: 10.1371/journal.pone.0118735 (PMC4370401; doi:10.1371/journal.pone.0118735)
Supplement: S4 Table — (PDF) [file pone.0118735.s007.pdf]

Table S4. Correlation between As and Fe, Mn, SO_4, NO_3, HCO_3, TOC, and CFU in the groundwater, collected from Barasat and Chakdaha, West Bengal.

	**Correlation (R^2 Value)**							
	**Fe**	**Mn**	**SO_4**	**NO_3**	**Depth**	**HCO_3**	**TOC**	**CFU**
**As**	0.029	0.27	0.13	0.014	0.62	0.61	0.41	0.041
