# Supplementary material for: Diversity, Metabolic Properties and Arsenic Mobilization Potential of Indigenous Bacteria in Arsenic Contaminated Groundwater of West Bengal, India
Source: PLoS One. 2015 Mar 23;10(3):e0118735. doi: 10.1371/journal.pone.0118735 (PMC4370401; doi:10.1371/journal.pone.0118735)
Supplement: S5 Table — (PDF) [file pone.0118735.s008.pdf]

Table S5. Designations of the isolates corresponding to the numbers allotted in the PCA plot (Fig. 9b)

No.	Strain ID	No.	Strain ID
1	*Acinetobacter* BAS123i	23	*Pseudomonas* CAS4001i
2	*Arthrobacter* CAS4117i	24	*Pseudomonas* BAS323i
3	*Arthrobacter* CAS4101i	25	*Pseudomonas* CAS907i
4	*Bacillus* BAS204i	26	*Rheinheimera* BAS124i
5	*Brevundimonas* BAS230i	27	*Rheinheimera* BAS122i
6	*Brevundimonas* CAS4119i	28	*Rheinheimera* BAS127i
7	*Brevundimonas* CAS4123i	29	*Rhizobium* CAS325i
8	*Brevundimonas* CAS4008i	30	*Rhizobium* BAS306i
9	*Brevundimonas* BAS223i	31	*Rhizobium* CAS4026i
10	*Brevundimonas* CAS4005i	32	*Rhizobium* CAS4022i
11	*Herbaspirillum* CAS4110i	33	*Rhizobium* BAS310i
12	*Hydrogenophaga* CAS4014i	34	*Rhizobium* BAS316i
13	*Microbacterium* CAS905i	35	*Rhizobium* BAS305i
14	*Phyllobacterium* BAS211i	36	*Rhodococcus* CAS912i
15	*Phyllobacterium* BAS224i	37	*Rhodococcus* CAS931i
16	*Pseudomonas* CAS934i	38	*Rhodococcus* CAS930i
17	*Pseudomonas* CAS4116i	39	*Rhodococcus* CAS933i
18	*Pseudomonas* CAS4106i	40	*Rhodococcus* CAS4021i
19	*Pseudomonas* CAS4105i	41	*Rhodococcus* CAS922i
20	*Pseudomonas* BAS309i	42	*Staphylococcus* CAS106i
21	*Pseudomonas* CAS4016i	43	*Staphylococcus* BAS108i
22	*Pseudomonas* CAS908i	44	*Stenotrophomonas* BAS202i
