# Supplementary material for: Diversity, Metabolic Properties and Arsenic Mobilization Potential of Indigenous Bacteria in Arsenic Contaminated Groundwater of West Bengal, India
Source: PLoS One. 2015 Mar 23;10(3):e0118735. doi: 10.1371/journal.pone.0118735 (PMC4370401; doi:10.1371/journal.pone.0118735)
Supplement: S6 Table — (PDF) [file pone.0118735.s009.pdf]

Table S6. Microbial counts during 300 days incubation in microcosm experiment (CFU/ml).

Days	Sample ID								
	**CAS922i**	**CAS4005i**	**BAS108i**	**BAS224i**	**CAS4101i**	**BAS323i**	**BAS123i**	**CAS907i**	**Control**
0	3.00E+06±0.7	7.30E+07±0.6	2.00E+06±0.5	3.30E+06±0.4	1.30E+06±0.7	4.60E+06±0.5	4.00E+06±0.8	2.00E+06±0.5	0
1	4.30E+07±0.2	9.70E+07±0.3	3.60E+07±0.7	3.80E+06±0.2	2.60E+07±0.8	9.40E+06±0.7	8.00E+06±0.7	6.00E+07±0.8	0
7	6.90E+07±0.5	8.60E+07±0.5	5.00E+07±0.8	5.30E+07±0.7	8.00E+07±0.9	9.00E+07±0.4	1.80E+08±0.4	6.60E+07±0.4	0
300	5.10E+07±0.8	3.40E+07±0.7	3.40E+07±0.7	3.10E+07±0.9	1.75E+07±0.4	5.10E+07±0.5	1.80E+07±0.5	4.10E+07±0.7	0

The isolates are denoted as follows: CAS922i (*Rhodococcus* sp.), CAS4005i (*Brevundimonas* sp.), BAS108i (*Staphylococcus* sp.), BAS224i (*Phyllobacterium* sp.), CAS4101i (*Arthrobacter* sp.), BAS323i (*Pseudomonas* sp.), BAS123i (*Acinetobacter* sp.) and CAS907i (*Pseudomonas* sp.).
