# Supplementary material for: Diversity, Metabolic Properties and Arsenic Mobilization Potential of Indigenous Bacteria in Arsenic Contaminated Groundwater of West Bengal, India
Source: PLoS One. 2015 Mar 23;10(3):e0118735. doi: 10.1371/journal.pone.0118735 (PMC4370401; doi:10.1371/journal.pone.0118735)
Supplement: S7 Table — (PDF) [file pone.0118735.s010.pdf]

Table S7. Elemental composition of the orange sand before and after 300 days incubation with selected bacterial strains under anaerobic condition (XRF analysis).

Elements	Sample ID									
	Original sediment	After 300 days incubation								
		Control	BAS108i	BAS123i	BAS323i	CAS907i	CAS922i	CAS4005i	CAS4101i	BAS224i
Al(%)	7.672	7.503	7.36	7.788	6.988	7.06	7.756	6.908	7.344	7.231
Fe(%)	3.615	3.452	2.846	3.102	3.468	3.131	3.064	3.32	3.146	3.583
S(%)	0.054	0.062	0.059	0.057	0.066	0.058	0.056	0.068	0.06	0.067
Mg(%)	1.373	1.252	1.142	1.263	1.18	1.128	1.245	1.116	1.18	1.235
Ca(%)	2.957	2.821	2.356	2.906	2.273	3.028	2.85	3.072	2.87	2.31
Na(%)	0.565	0.699	0.87	0.874	0.586	0.737	0.845	0.758	0.696	0.661
K(%)	2.215	2.209	2	2.135	2.081	2.101	2.127	2.067	2.097	2.115
Mn(%)	0.061	0.058	0.054	0.056	0.059	0.056	0.056	0.058	0.056	0.06
Ti(%)	0.556	0.554	0.434	0.476	0.533	0.484	0.471	0.525	0.5	0.544
P(%)	0.038	0.046	0.034	0.043	0.03	0.037	0.044	0.038	0.041	0.034
Ba(ppm)	345.76	331.33	261.27	280.26	318.97	291.30	280.92	312.71	301.67	329.86
Cr(ppm)	107.93	124.50	89.101	104.69	114.01	106.29	90.023	108.01	106.25	135.69
Co(ppm)	11.641	11.517	10.512	10.975	11.704	10.999	10.961	11.22	10.98	11.887
Cu(ppm)	72.626	77.479	77.825	75.834	77.175	78.212	74.714	74.728	75.709	81.765
Br(ppm)	13.653	14.441	29.358	22.036	22.647	20.618	24.694	18.642	18.02	19.805
La(ppm)	40.62	40.506	40.1	38.1	43.416	40.149	39.661	41.108	39.775	41.4
Ce(ppm)	81.896	83.05	83.439	84.013	82.422	83.317	83.965	82.306	82.92	82.149
Eu(ppm)	0.992	0.984	0.98	0.983	0.99	0.983	0.982	0.986	0.983	0.989
Hf(ppm)	9.456	9.7	13.642	11.12	11.427	11.037	11.88	10.591	10.884	10.967
Mo(ppm)	11.53	11.225	15.02	13.397	13.137	12.827	14.11	12.393	12.357	12.522
Nb(ppm)	17.942	17.772	20.468	19.266	19.171	18.849	19.725	18.584	18.446	18.844
Ni(ppm)	89.147	86.699	91.89	90.75	92.438	87.726	90.019	90.188	90.595	93.31
Zn(ppm)	142.567	132.053	134.246	134.032	135.945	134.18	135.163	133.712	131.044	137.489
Rb(ppm)	160.035	163.139	169.956	163.876	176.225	161.216	167.544	161.259	161.783	176.724
Sr(ppm)	414.445	409.009	435.575	429.861	419.007	424.093	432.401	421.74	425.342	415.444
Zr(ppm)	269.021	257.48	277.848	282.24	264.177	279.937	280.578	267.269	271.277	257.783
Sm(ppm)	5.785	5.719	5.737	5.635	5.954	5.715	5.749	5.688	5.633	5.863
Pb(ppm)	38.673	36.95	41.944	39.593	40.218	38.669	40.415	39.523	38.575	39.569
Th(ppm)	18.548	18.219	21.566	20.16	19.928	19.73	20.716	19.232	19.32	19.758
Sc(ppm)	13.012	13.165	12.619	11.749	13.328	12.764	12.09	12.56	12.636	13.763
V(ppm)	92.079	91.071	76.929	81.445	90.804	84.298	79.418	87.418	85.111	90.027

The isolates are denoted as follows: CAS922i (*Rhodococcus* sp.), CAS4005i (*Brevundimonas* sp.), BAS108i (*Staphylococcus* sp.), BAS224i (*Phyllobacterium* sp.), CAS4101i (*Arthrobacter* sp.), BAS323i (*Pseudomonas* sp.), BAS123i (*Acinetobacter* sp.) and CAS907i (*Pseudomonas* sp.).
