# Supplementary material for: Diversity, Metabolic Properties and Arsenic Mobilization Potential of Indigenous Bacteria in Arsenic Contaminated Groundwater of West Bengal, India
Source: PLoS One. 2015 Mar 23;10(3):e0118735. doi: 10.1371/journal.pone.0118735 (PMC4370401; doi:10.1371/journal.pone.0118735)
Supplement: S8 Table — (PDF) [file pone.0118735.s011.pdf]

Table S8. Correlation between As and Mn, Fe, Na, Ca, K and Al in the aqueous phase of microcosm after 300 days incubation

	**Correlation (R^2 Value)**					
	**Mn**	**Fe**	**Na**	**Ca**	**K**	**Al**
**As**	0.15	0.114	0.404	0.258	0.056	0.044
